# Quantum refinement with multiple conformations: application to the P-cluster in nitrogenase

**DOI:** 10.1107/S2059798320012917

**Published:** 2020-10-16

**Authors:** Lili Cao, Ulf Ryde

**Affiliations:** a Department of Theoretical Chemistry, Lund University, PO Box 124, 221 00 Lund, Sweden

**Keywords:** crystallographic refinement, quantum refinement, multiple conformations, nitrogenase, P-cluster

## Abstract

X-ray crystallography is the main source of atomistic information on the structure of proteins. Normal crystal structures are obtained as a compromise between the X-ray scattering data and a set of empirical restraints that ensure chemically reasonable bond lengths and angles. However, such restraints are not always available or accurate for nonstandard parts of the structure, for example substrates, inhibitors and metal sites. The method of quantum refinement, in which these empirical restraints are replaced by quantum-mechanical (QM) calculations, has previously been suggested for small but interesting parts of the protein. Here, this approach is extended to allow for multiple conformations in the QM region by performing separate QM calculations for each conformation. This approach is shown to work properly and leads to improved structures in terms of electron-density maps and real-space difference density *Z*-scores. It is also shown that the quality of the structures can be gauged using QM strain energies. The approach, called *ComQumX*-2*QM*, is applied to the P-cluster in two different crystal structures of the enzyme nitrogenase, *i.e.* an Fe_8_S_7_Cys_6_ cluster, used for electron transfer. One structure is at a very high resolution (1.0 Å) and shows a mixture of two different oxidation states, the fully reduced P^N^ state (Fe_8_^2+^, 20%) and the doubly oxidized P^2+^ state (80%). In the original crystal structure the coordinates differed for only two iron ions, but here it is shown that the two states also show differences in other atoms of up to 0.7 Å. The second structure is at a more modest resolution, 2.1 Å, and was originally suggested to show only the one-electron oxidized state, P^1+^. Here, it is shown that it is rather a 50/50% mixture of the P^1+^ and P^2+^ states and that many of the Fe—Fe and Fe—S distances in the original structure were quite inaccurate (by up to 0.8 Å). This shows that the new *ComQumX*-2*QM* approach can be used to sort out what is actually seen in crystal structures with dual conformations and to give locally improved coordinates.

## Abstract 2

The method of quantum refinement has been extended to systems with multiple conformations in the quantum system. It has been used to improve the description of the P-cluster in three different oxidation states in two crystal structures of the enzyme nitrogenase.

## 1. Introduction   

X-ray crystallography is currently the prime method for obtaining atomic-resolution structural information on bio­logical macromolecules. Such information has been crucial for our understanding of the function of these molecules, opening up the rational construction of enzymes with new functions and the design of new drugs. However, the approach has some limitations. Firstly, H atoms scatter so weakly that they are normally not discerned, except at very high resolution. Therefore, protonation and tautomeric states cannot be decided from the structures, which is unfortunate because they are often crucial for understanding function.

Secondly, the resolution of the structures is typically limited, meaning that the exact positions of the atoms are not precisely defined, so that bond lengths and angles, if freely refined, may become somewhat strange. This is normally solved by adding some chemical information to the structure in the form of empirical bond-length, angle and dihedral restraints. In terms of computational chemistry, this is a molecular-mechanics potential, although in crystallography it is normally based on a statistical analysis of high-resolution crystal structures (Engh & Huber, 1991 ▸), rather than on energetic considerations.

Thus, the crystal structure is obtained as a compromise between the experimental data and the empirical potential by minimizing a target function of the form 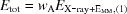
where *E*_X-ray_ is the experimental target function (describing how well the current model reproduces the experimental data; typically a maximum-likelihood function; Pannu & Read, 1996 ▸; Adams *et al.*, 1997 ▸) and *E*_MM_ is the empirical potential. *w*_A_ is a weight factor that is needed because the two terms do not have the same units and it determines the relative importance of the two terms. It is normally determined to give MM and crystallographic forces of an equal magnitude in a short molecular-dynamics simulation of the system (Brünger & Rice, 1997 ▸; Brünger *et al.*, 1989 ▸; Adams *et al.*, 1997 ▸).

This means that essentially all crystal structures are not purely experimental, but contain a significant aspect of molecular modelling. Moreover, the final structure depends on the quality of the empirical potential, which may differ for different parts of the structure. For proteins and DNA the potential is quite reliable, because a large amount of accurate structural information is available. However, for substrates, inhibitors and nonstandard residues, the potential may be much worse because less experimental information is available. For metal sites the situation is even worse, because metal sites are hard to model with an empirical (or molecular-mechanics) potential (Hu & Ryde, 2011 ▸).

Thirdly, X-ray crystallography also has problems in discerning differences between atoms containing one electron more or less. This means that it is often not possible to decide the oxidation states of atoms, *e.g.* of metal ions. This problem is further complicated by the fact that the oxidation state often changes during data collection owing to photoreduction by electrons released by the X-ray beam.

To address these problems, we have developed an approach called quantum refinement (Ryde *et al.*, 2002 ▸). In this, we replace the empirical potential in (1) by more accurate quantum-mechanical (QM) calculations for a small but interesting part of the macromolecule, for example an enzyme active site (called system 1 in the following). This gives the target function 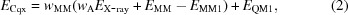
where *E*_QM1_ is the QM energy of QM system 1, *E*_MM_ is the empirical potential of everything (*i.e.* the same as in equation 1) and *E*_MM1_ is the empirical energy of the QM system (which is needed to avoid double counting of this energy). *w*_MM_ is a weighting function that is needed because the empirical potential in crystallo­graphic refinement software is based on statistics rather than energies (as is the QM term). This is analogous to standard QM/MM calculations in computational chemistry (Senn & Thiel, 2009 ▸; Ryde, 2016 ▸) employing a subtractive QM/MM approach (Cao & Ryde, 2018 ▸). We have shown that quantum refinement can locally improve crystal structures (Ryde & Nilsson, 2003 ▸), decide the protonation state of metal-bound ligands (Nilsson & Ryde, 2004 ▸; Cao *et al.*, 2017 ▸; Cao, Caldararu & Ryde, 2018 ▸; Caldararu *et al.*, 2018 ▸) and the oxidation state of metal sites (Rulíšek & Ryde, 2006 ▸; Cao *et al.*, 2019 ▸) and protein ligands (Caldararu *et al.*, 2018 ▸), detect the photoreduction of metal ions (Nilsson *et al.*, 2004 ▸; Söderhjelm & Ryde, 2006 ▸; Rulíšek & Ryde, 2006 ▸) and solve scientific problems regarding what is really seen in crystal structures (Söderhjelm & Ryde, 2006 ▸; Cao, Caldararu, Rosenzweig *et al.*, 2018 ▸; Nilsson *et al.*, 2004 ▸). Several other groups have implemented this and similar approaches, for example involving QM calculations of the entire crystal structure (Yu *et al.*, 2005 ▸; Borbulevych *et al.*, 2014 ▸; Hsiao *et al.*, 2010 ▸; Zheng, Reimers *et al.*, 2017 ▸; Zheng, Moriarty *et al.*, 2017 ▸; Genoni *et al.*, 2018 ▸).

However, a major problem with quantum refinement has been that it could not deal with multiple conformations within the QM system. Multiple conformations are frequently seen in crystal structures, especially for metal sites, either because the original sample is not in a pure state or owing to photoreduction during data collection. Quantum refinement is a powerful technique for detecting the presence of multiple conformations, characterized by the fact that no single structural interpretation fits the crystallographic data satisfactorily (Söderhjelm & Ryde, 2006 ▸; Rulíšek & Ryde, 2006 ▸; Cao *et al.*, 2019 ▸; Cao & Ryde, 2020 ▸). For example, we have shown that a recent crystal structure of the putative one-electron oxidized state (P^1+^) of the P-cluster in nitrogenase (Keable *et al.*, 2018 ▸) actually contains a mixture of one- and two-electron oxidized states (Cao *et al.*, 2019 ▸). This means that the structural details (for example Fe—Fe and Fe—S bond lengths) of the crystal structure cannot be trusted, even after quantum refinement, because the structure is a mixture of two oxidation states, giving distances that are weighted averages.

Here, we suggest a solution for this problem by implementing quantum refinement with multiple conformations in the QM system. Such an approach requires two QM calculations, one of each state, but the implementation is otherwise straightforward. We apply it to the P-cluster in nitrogenase both for the abovementioned mixture of the one- and two-electron oxidized states and for another crystal structure at a higher resolution, showing a mixture of the fully reduced (P^N^) and two-electron oxidized (P^2+^) states (Spatzal *et al.*, 2011 ▸).

## 2. Methods   

### 2.1. Crystal structures and quantum refinement   

The quantum-refinement calculations were performed on two crystal structures of nitrogenase. The first is an atomic-resolution structure (1.0 Å) of nitrogenase with the P-cluster in a mixture of the P^N^ and P^2+^ states (PDB entry 3u7q; Spatzal *et al.*, 2011 ▸). The second is the recent crystal structure of the putative P^1+^ state of the P-cluster at 2.10 Å resolution (PDB entry 6cdk; Keable *et al.*, 2018 ▸). Coordinates, occupancies, *B* factors and structure factors were obtained from the PDB files 3u7q and 6cdk. From these files, we also obtained the space group, unit-cell parameters, resolution limits, *R* factors and the test set used for evaluation of the *R*_free_ factor.

In the quantum-refinement calculations, we employed the *Crystallography and NMR System* (*CNS*) software (Brünger *et al.*, 1998 ▸; Brunger, 2007 ▸) version 1.3 for the crystallographic calculations. The full protein was used in all calculations, including all crystal water molecules. All alternative conformations in the original crystal structure (PDB entry 3u7q) were also considered. For the protein, we used the standard *CNS* force field (protein_rep.param, water_rep.param and ion.param; Brünger *et al.*, 1998 ▸; Brunger, 2007 ▸). The metal sites were treated as individual isolated ions, whereas the MM force fields for other nonstandard residues were downloaded from the Hetero-compound Information Centre Uppsala (Kleywegt, 2007 ▸). The *w*_A_ factor was determined by *CNS* to be 3.68 and 0.0794 for PDB entries 6cdk and 3u7q, respectively. The *w*_MM_ weight was set to 1/3 as in all our previous studies (Ryde *et al.*, 2002 ▸; Cao *et al.*, 2019 ▸). For the crystallographic target function, we used the standard maximum-likelihood function using amplitudes (mlf) in *CNS* (Pannu & Read, 1996 ▸; Adams *et al.*, 1997 ▸). *CNS* does not support anisotropic *B* factors, which were present in the structure with PDB code 3u7q. Therefore, only isotropic *B* factors were used. However, after the quantum refinement, anisotropic *B*-factor (and sometimes also occupancy) refinement was performed using *phenix.refine* (Afonine *et al.*, 2012 ▸). Electron-density maps were generated using *phenix.maps* (Afonine *et al.*, 2012 ▸).

The quality of the models was compared using the real-space difference density *Z*-score (RSZD; Tickle, 2012 ▸) calculated by *EDSTATS* (part of the *CCP*4 package), which measures the local accuracy of the model. The maximum of the absolute values of the positive and negative RSZD (combined RSZD) was calculated for the whole P-cluster, together with the six cysteine ligands and the neighbouring GlyC87 and SerD188 (C and D in the residue numbers indicate that the residues belong to the C and D subunits of the protein). RSZD should typically be less than 3.0 for a good model.

### 2.2. QM calculations   

All QM calculations were performed with the *TURBOMOLE* software (version 7.3; Furche *et al.*, 2014 ▸). We employed the meta generalized gradient approximation functional TPSS (Tao *et al.*, 2003 ▸) and the def2-SV(P) basis sets (Schäfer *et al.*, 1992 ▸). This level of theory has been shown to give excellent geometries for both the FeMo and P-clusters in nitrogenase (Cao & Ryde, 2019 ▸; Cao *et al.*, 2019 ▸). The calculations were sped up by expanding the Coulomb interactions in an auxiliary basis set: the resolution-of-identity approximation (Eichkorn *et al.*, 1995 ▸, 1997 ▸). Empirical dispersion corrections were included with the DFT-D3 approach (Grimme *et al.*, 2010 ▸) with Becke–Johnson damping (Grimme *et al.*, 2011 ▸).

**Figure 1:**
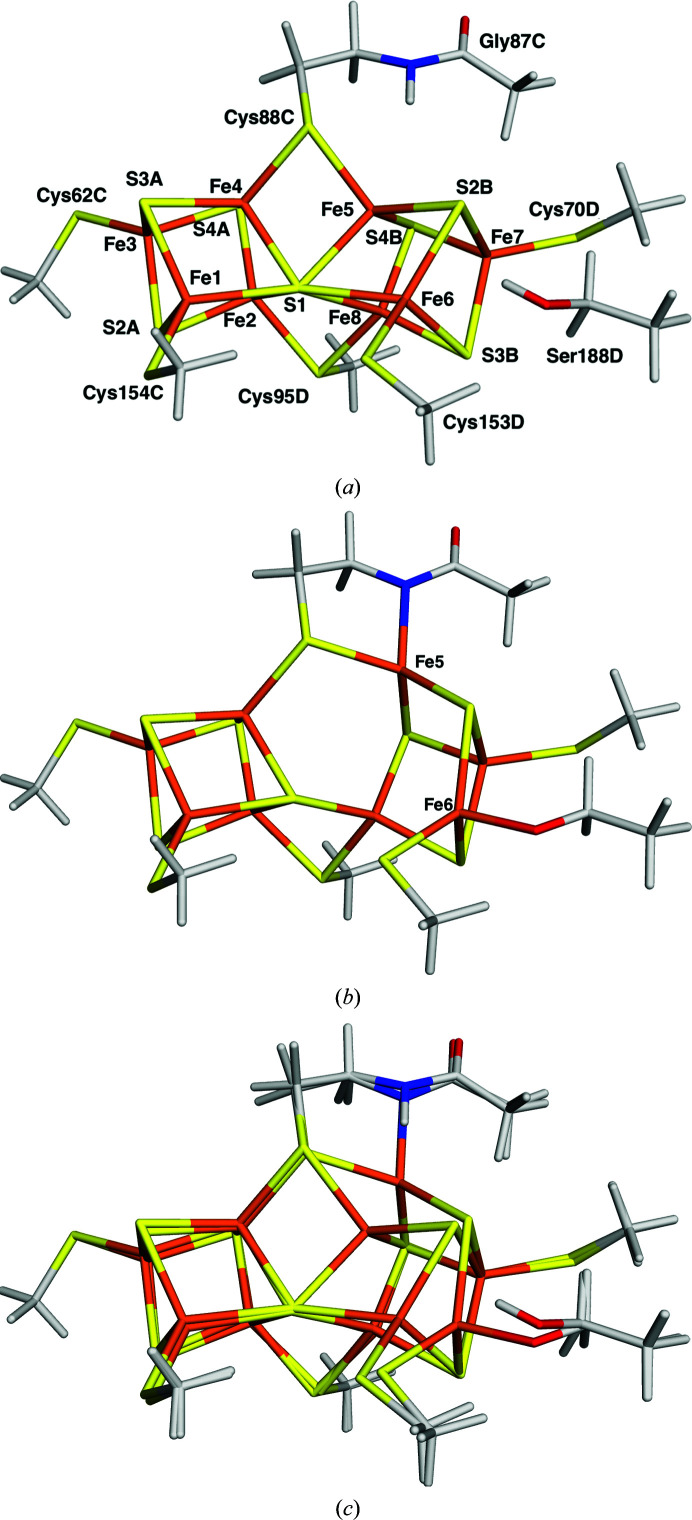
The *ComQumX*-2*QM* structures (*w*_A_ = 0.079) from PDB entry 3u7q for the (*a*) P^N^ and (*b*) P^2+^ states, showing the QM system employed in all calculations, the numbering of the protein residues and the naming of the Fe and S atoms. (*c*) shows an overlay of the two structures.

The QM system consisted of the full P-cluster bound between the C and D subunits of nitrogenase with all coordinating cysteine residues modelled by CH_3_S^−^. In addition, a CH_3_OH model of SerD188 was included and CysC88 was modelled by CH_3_CONHCH_2_CH_2_S^−^, *i.e.* including the backbone between GlyC87 and CysC88. The QM system is illustrated in Fig. 1 ▸. Bonds between the QM system and the surrounding protein were treated by the hydrogen link-atom approach (Reuter *et al.*, 2000 ▸; Senn & Thiel, 2009 ▸; Ryde, 2016 ▸) as implemented in the *ComQum* software (Ryde, 1996 ▸; Ryde & Olsson, 2001 ▸).

In the P^N^ state, the backbone N atom of CysC88 and the side chain of SerD188 were both protonated. Thus, the model was Fe_8_S_7_(CH_3_S)_5_(CH_3_CONHCH_2_CH_2_S)(CH_3_OH) with a net charge of −4 (Fig. 1 ▸ *a*). In the P^1+^ state, SerD188 was deprotonated and CysC88 was protonated. In the P^2+^ state, both CysC88 and SerD188 were deprotonated, *i.e.* the model was Fe_8_S_7_(CH_3_S)_5_(CH_3_CONCH_2_CH_2_S)(CH_3_O) (Fig. 1 ▸ *b*). These have been shown to be the proper protonation states of the P-cluster (Keable *et al.*, 2018 ▸; Cao *et al.*, 2019 ▸). Thereby, the net charge of the QM system is −4 in all three states, because the addition of an electron is compensated by the deprotonation of SerD188 or CysC88.

In analogy with standard crystallographic refinement, no electrostatics were included in the MM calculations (because the positions of the H atoms are not discerned). However, in the QM calculations H atoms are present and electrostatics are of course considered.

All QM calculations of the P-cluster were based on the broken-symmetry (BS) approach (Noodleman, 1981 ▸; Lovell *et al.*, 2001 ▸). Thus, each Fe ion is in the high-spin (*S* = 2 or 5/2) state, but the spins couple antiferromagnetically to give a lower net spin. Following our previous thorough study of all possible BS states (Cao *et al.*, 2019 ▸), we used the BSb11 state (*i.e.* with minority spin on the Fe1, Fe2, Fe4 and Fe7 ions) for the P^N^ and P^1+^ states, and BSc35 with minority spin on Fe3, Fe5 and Fe8 for the P^2+^ state. We studied the experimentally observed spin states: *S* = 0, 1/2 and 4 for the P^N^, P^1+^ and P^2+^ states, respectively (Mouesca *et al.*, 1994 ▸; Tittsworth & Hales, 1993 ▸). A starting wavefunction with the correct spin and BS state was obtained by the fragment approach of Szilagyi & Winslow (2006 ▸).

## 3. Results and discussion   

We have extended the quantum-refinement approach to allow for multiple conformations in the QM systems. Below, we first describe the implementation of this approach with dual conformations and then illustrate its performance for the P-cluster in two crystal structures of nitrogenase: PDB entries 3u7q (Spatzal *et al.*, 2011 ▸) and 6cpk (Keable *et al.*, 2018 ▸).

### 3.1. Implementation   

As already mentioned in Section 1, the philosophy of quantum refinement is to replace the empirical restraints used in standard crystallographic refinement to ensure that the final structure makes chemical sense (*i.e.* it has reasonable bond lengths and angles) with more accurate QM calculations. Thereby, the resulting structure will be an optimum compromise between the crystallographic raw data and the QM energy function. Therefore, we only introduce QM calculations in the final steps of the structure-determination process, typically after a standard structure refinement. This will not affect the overall structure, but it will provide a better geometry of the QM system, which may help to interpret ambiguous electron-density maps and to decide what is really seen in the crystal structure.

We selected the *Crystallography and NMR System* (*CNS*) software (Brünger *et al.*, 1998 ▸; Brunger, 2007 ▸) for several reasons. Firstly, *CNS* is freely available software for X-ray crystallographic refinement. Secondly, our previous implementations of quantum-refinement methods used the *CNS* software (Ryde *et al.*, 2002 ▸; Caldararu *et al.*, 2019 ▸), which made extension to two QM systems straightforward. Thirdly, *CNS* was originally developed from the *CHARMM* MM software (Brooks *et al.*, 2009 ▸), meaning that it consists of an open symbolic language with existing implementation of MM force fields, with facile access to and manipulation of energies and forces, again strongly simplifying the implementation.

Crystallographic refinement is in principle a global pseudo-energy minimization using the energy function in (1),[Disp-formula fd1] and the energy function of standard quantum refinement is given by (2). If there are dual conformations in the QM system, separate calculations need to be employed for the two alternative conformations of the QM system, 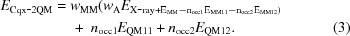
Here, *E*_X-ray_ and *E*_MM_ are the same as in (2), but they now involve alternative conformations of atoms in the QM system, treated with standard methods in the crystallography software (for example to avoid interactions between the two conformations in the MM term). *E*_QM11_ and *E*_MM11_ are the QM and MM energies of the first conformation of the QM system (called system 11), which has occupancy *n*_occ1_. Likewise, *E*_QM12_ and *E*_MM12_ are the QM and MM energies of the second conformation of the QM system (called system 12), which has occupancy *n*_occ2_. Forces are obtained from this energy function by using analytical differentiation and employing the chain rule for the hydrogen link-atoms (Ryde, 1996 ▸; Ryde & Olsson, 2001 ▸).

**Figure 2:**
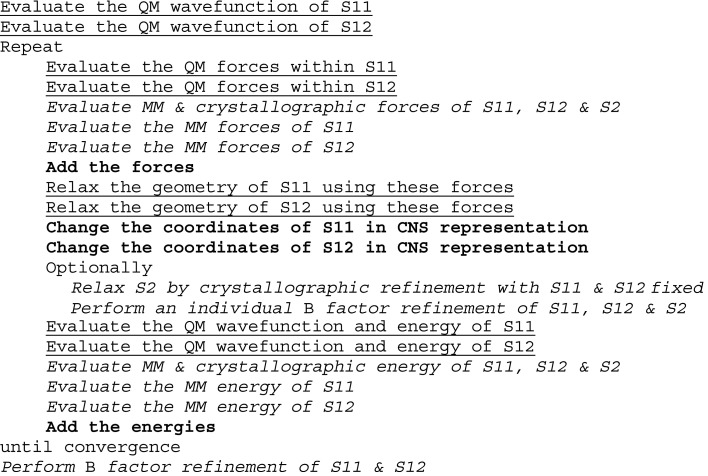
Flow chart of the *ComQumX*-2*QM* program. S11, S12 and S2 denote systems 11, 12 and 2 (system 2 is all atoms outside systems 11 and 12). Steps in bold constitute the actual *ComQumX*-2*QM* interface. Steps in italics are performed by the crystallographic refinement program, whereas those underlined are run by the QM program. The whole procedure is driven by a Linux shell script.

The flow of the new program, called *ComQumX*-2*QM*, is shown in Fig. 2 ▸. It is similar to that of standard quantum refinement, but every step involving system 1 has been duplicated to perform calculations for both systems 11 and 12. Moreover, the calculations of the joint energies and forces have been updated to take into account the two QM systems and the occupancies. The implementation uses the *CNS* minimize.inp and bindividual.inp files, with some simple modifications to write out crystallographic energies and forces. This ensures that all normal crystallographic manipulations and calculations are properly performed, for example bulk-solvent corrections and calculations of the *R* factors. Moreover, the files are changed to read in and write out coordinates with a higher precision than standard PDB files to avoid convergence problems (Ryde *et al.*, 2002 ▸). For the crystallographic energy and force calculations, the number of minimization steps was set to zero, whereas when the coordinates and *B* factors of the surrounding protein are refined it was set to unity. A simple *CNS* script was also set up to calculate the *E*_MM11_ and *E*_MM12_ energy terms. The whole quantum-refinement procedure is driven by a Linux shell script, which is based on the *TURBOMOLE* geometry-optimization script *jobex* (Furche *et al.*, 2014 ▸). The relaxation of the QM system is performed by the *relax* program in *TURBOMOLE*, employing a Broyden–Fletcher–Goldfarb–Shanno quasi-Newton method. Further descriptions of the procedure and the setup of the calculations can be found at http://signe.teokem.lu.se/~ulf/Methods/comqumx-2qm.html and the interface can be provided by the authors upon request (however, *CNS* and *TURBOMOLE* need to be installed separately and a licence for *TURBOMOLE* is required).

The *ComQumX*-2*QM* approach is somewhat similar to the *QM/MM*-2*QM* method (Hu *et al.*, 2011 ▸). However, in the latter both QM systems are fully occupied and located at different positions in the biomacromolecule. It is primarily intended to study electron and proton transfer.

### 3.2. Performance of *ComQumX*-2*QM* for PDB entry 3u7q   

The *ComQumX*-2*QM* approach has been applied to the P-cluster in nitrogenase based on two different crystal structures involving two different sets of oxidation states. The first, PDB entry 3u7q (Spatzal *et al.*, 2011 ▸), is an atomic resolution structure (1.0 Å resolution) of the resting state of the enzyme. In the original crystal structure, two conformations were reported for the P-cluster, which were interpreted as the fully reduced state (P^N^, *i.e.* with all eight iron ions in the Fe^2+^ state; 20% occupancy) and the two-electron oxidized state (P^2+^; 80% occupancy). However, in practice the coordinates of only two atoms (Fe5 and Fe6) differed between the two conformations (by 1.2–1.3 Å), whereas all of the other coordinates were identical. Therefore, the structure of the P-cluster cannot be expected to be as accurate as the other parts of the crystal structure. Instead, the other atoms in the P-cluster have positions that are a compromise between the preferences in the two oxidation states, as has been discussed before (Cao *et al.*, 2019 ▸). In fact, the QM/MM structures of the P^N^ and P^2+^ states had maximum deviations of the Fe—Fe and Fe—S bond lengths from the crystal structure that were 2–4 times larger than for the active-site FeMo cluster: 0.17–0.25 Å compared with 0.06–0.09 Å.

The two-electron oxidation of the P-cluster from P^N^ to P^2+^ is accompanied by the deprotonation of two protein residues: the OG atom of SerD188 and the backbone N atom of CysC88 (Spatzal *et al.*, 2011 ▸; Cao *et al.*, 2019 ▸; Keable *et al.*, 2018 ▸). The deprotonated groups coordinate to the Fe6 and Fe5 ions, causing the movement of these two ions away from the S1 ion, cleaving the Fe5—S1 and Fe6—S1 bonds and giving rise to a distorted structure of the second cubane in the P-cluster in the P^2+^ state.

The structures of the P^N^ (20% occupancy) and P^2+^ (80% occupancy) states from *ComQumX*-2*QM* quantum refinement with the default weight factor of *w*_A_ = 0.079 are shown in Figs. 1 ▸(*a*) and 1 ▸(*b*). It can be seen that they show the expected geometries. P^N^ has two regular cubane clusters connected at the S1 sulfide ion and with two bridging Cys residues. Neither the protonated OG atom of SerD118 nor the protonated backbone N atom of CysC88 coordinate to their Fe ions (Fe6–OG distance of 3.57 Å and Fe5–N distance of 3.48 Å). In the P^2+^ state, the second cubane cluster (involving Fe5–Fe8) is distorted with a missing sulfide corner. The deprotonated OG atom of SerD118 forms a strong bond to Fe6 with an Fe–O distance of 1.89 Å and the deprotonated backbone N atom of CysC88 coordinates to Fe5 with an Fe—N bond length of 2.08 Å.

However, in contrast to the original structure, the change in oxidation state causes movements of all atoms in the cluster, rather than of only the Fe5 and Fe6 ions, as can be seen in Fig. 1 ▸(*c*). The latter two ions show the largest movements: 1.37 and 1.33 Å, respectively. However, the OG atom of SerD188 moves by 0.70 Å, the S atom of CysD153 moves by 0.48 Å, S2B (bridging Fe5 and Fe6) moves by 0.33 Å, the N atom of CysC88 moves by 0.28 Å and Fe8 moves by 0.24 Å. On the other hand, no atom in the first cubane (involving Fe1–Fe4) moves by more than 0.2 Å between the two oxidation states. Interestingly, Fe7 is essentially also fixed (movement of 0.02 Å). This illustrates the level of approximation used when the two oxidation states in the original crystal structure were modelled using distinct coordinates for only Fe5 and Fe6. Still, this was necessary in the crystal structure because no accurate force field (or empirical restraints) is available for the P-cluster. The QM calculations in our quantum refinement solve this problem.

The quantum-refined structure is quite close to the original crystallographic coordinates (PDB entry 3u7q). The P^2+^ state reproduces the Fe–Fe distances with a mean absolute deviation (MAD) of 0.02 Å and a maximum deviation of 0.05 Å for the Fe6–Fe8 distance, whereas the Fe—S bonds are reproduced with a MAD of 0.01 Å and a maximum deviation of 0.05 Å for the Fe6–SG_153D_ distance. For the P^N^ state the deviations are larger: the MAD for the Fe–Fe distances is 0.04 Å, with a maximum deviation of 0.12 Å for the Fe6–Fe8 distance, whereas the MAD for the Fe—S bonds is 0.06 Å, with a maximum deviation of 0.21 Å for the Fe5—S1 bond. This reflects that the coordinates of the P-cluster and its ligands (besides Fe5 and Fe6) are a weighted average of the P^N^ and P^2+^ structures, and that the weight (the occupancy) is four times larger for P^2+^ than for P^N^. Undoubtedly, the coordinates are more accurate in the quantum-refined structure (especially for P^N^).

The quantum-refined structures are also very similar to our previous QM/MM structures of the P^N^ and P^2+^ states in the same protein (Cao *et al.*, 2019 ▸). The MAD between the coordinates is 0.10 and 0.11 Å for the P^N^ and P^2+^ states, respectively, with maximum differences of 0.23 and 0.19 Å, respectively. This illustrates that the quantum-refined structures are mainly QM/MM structure at the locations where the experimental electron density cannot really discern between the two states.

Next, we investigated how the quantum-refined structures vary with the weight factor *w*_A_, which determines the relative importance of the crystallographic data and the empirical and QM restraints. The results are shown in Table 1 ▸. It can be seen that the crystallographic *R* factor decreases slightly as *w*_A_ is increased (except at the largest *w*_A_ of 0.3), reflecting that the weight of the crystallographic penalty function is increased. However, the change is minimal, 0.1437–0.1442, reflecting that we change the coordinates of only a very small fraction of the atoms. At *w*_A_ values larger than 0.3 convergence problems arise, which are often observed in quantum refinement when the restraints towards the crystal structure are too large to give a reasonable QM structure. At *w*_A_ = 0 the crystallographic penalty has been turned off, resulting in a pure QM/MM structure (but with the *CNS* force field, rather than the AMBER force field used in our previous QM/MM study; Cao *et al.*, 2019 ▸). This is reflected in a somewhat larger increase in the *R* factor of 0.1480. However, the restricted increase shows that the QM calculations actually give a quite realistic structure of the P-cluster (compared with the raw crystallographic data).

The *R*_free_ factor shows similar trends and gives the lowest value for *w*_A_ = 0.1 and 0.3. The variation is still minimal, 0.1601–0.1604, but is 0.1642 for *w*_A_ = 0. The difference between the two *R* factors indicates the overfitting and actually increases slightly with *w*_A_.

The two *R* factors are global measures that change minimally when the description of only the P-cluster changes. The RSZD score gives a better indication of local changes and is currently considered as the best measure to describe the local quality of a crystal structure (Tickle, 2012 ▸). We have therefore also calculated the RSZD score for the P-cluster (Fe and sulfide ions, maximum value for the P^N^ and P^2+^ conformations; the raw data are shown in Supplementary Table S1, which also gives the results for the coordinating residues). It can be seen that it decreases strongly with *w*_A_, from 100 for *w*_A_ = 0 to 9.1 for *w*_A_ = 0.3, with a slight minimum at *w*_A_ = 0.1.

Table 1 ▸ also shows the strain energy of the two QM systems. We define it as the difference in the energy of the two QM systems compared with that obtained with *w*_A_ = 0, *i.e.* the ideal QM/MM structure without any crystallographic restraints. It increases with *w*_A_, as expected, up to 31 and 25 kJ mol^−1^ at *w*_A_ = 0.3. Except for the largest *w*_A_ value, the strain is always larger for P^2+^ than for P^N^, reflecting the higher occupancy for the former (80%), indicating a larger restraint towards the crystal structure. The strain energy is a measure of the misfit between the preferences of QM and crystallography. At the resolution of the crystal structure with PDB code 3u7q (1.0 Å) a significant strain energy is expected, reflecting that the crystal structure is so accurate that small systematic errors in the DFT calculations become apparent. However, there are also statistical and phase errors in the crystal structure. Therefore, as in standard crystallographic refinement, the optimum structure is a compromise between the crystallo­graphic restraints and the QM and MM restraints obtained at intermediate values of *w*_A_. *CNS* suggests a value of *w*_A_ = 0.0794, whereas the best structure in terms of the RSZD score was obtained with *w*_A_ = 0.1.

To decide on an ideal *w*_A_ value, we also look at the electron-density maps. PDB entry 3u7q is an unusually accurate structure (1.0 Å resolution), meaning that essentially all atoms are visible in the 2*mF*_o_ − *DF*_c_ map at a 6σ level, as is shown in Supplementary Fig. S1. The two positions of Fe5 and Fe6 are also clearly discerned, together with the lower occupancy of the P^N^ conformation, although it appears that the alternative P^N^ conformation of Fe5 has a somewhat lower occupancy than that of Fe6 (maxima at 7.0σ and 7.6σ).

**Figure 3:**
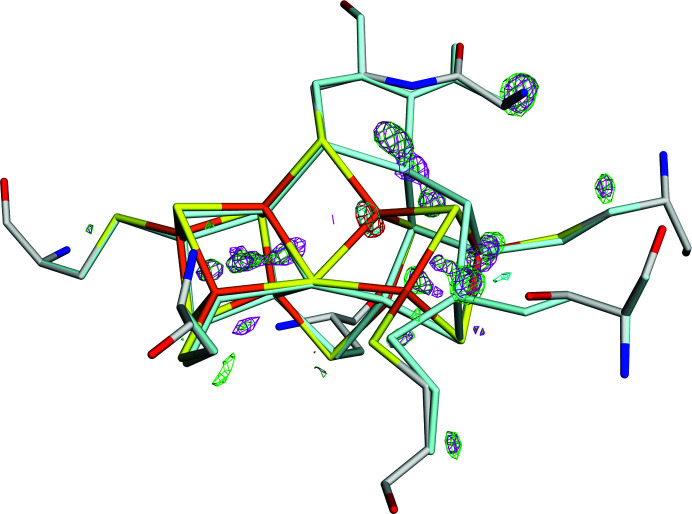
The *mF*_o_ − *DF*_c_ electron-density difference maps at a ±4σ level of the quantum-refined structures with *w*_A_ = 0.1 (positive, green; negative, red) and *w*_A_ = 0.3 (positive, magenta; negative, cyan) of the structure with PDB code 3u7q. The P^N^ state is shown in atomic colours, whereas the P^2+^ state is shown in pale cyan.

The *mF*_o_ − *DF*_c_ difference maps obtained from quantum refinement with *w*_A_ = 0.1 and 0.3 are shown in Fig. 3 ▸. The negative densities are closely similar for the two structures, with only a single prominent feature close to the P^N^ position of Fe5 (*w*_A_ = 0.3 gives an additional minimal feature close to Fe8). However, the volumes of positive density for *w*_A_ = 0.3 (magenta in Fig. 3 ▸) are appreciably larger than those for *w*_A_ = 0.1 (green), especially around Fe5. Therefore, we selected *w*_A_ = 0.1 as our best quantum-refined structure, a selection that is also supported by the lower strain energies, 10 and 24 kJ mol^−1^.

With the value of *w*_A_ = 0.1, we next ran an occupancy refinement of the P-cluster and the coordinating residues (*i.e.* those in the QM system shown in Fig. 1 ▸) using *Phenix* (so that anisotropic *B* factors are also considered). It showed that the original occupancies (20% for P^N^ and 80% for P^2+^) are close to optimal (we obtained average occupancies for the eight residues and the cluster of 19% and 81% for P^N^ and P^2+^, respectively). However, to visualize the effect of the occupancies, we ran two additional quantum-refinement calculations with *w*_A_ = 0.1: one with occupancies of 10% and 90% and the other with occupancies of 30% and 70% for P^N^ and P^2+^, respectively. The results of these refinements are also included in the lower part of Table 1 ▸. It can be seen that both *R* factors show only a minimal variation, but *R*_free_ is actually best for the calculation with occupancies of 30% and 70%. On the other hand, the RSZD score for the P-cluster deteriorates greatly with these occupancies and is actually lowest for the 10% and 90% occupancy calculation (5 compared with 25). However, the situation becomes more complicated if the RSZD scores of the coordinating residues are also considered (Supplementary Table S1).

**Figure 4:**
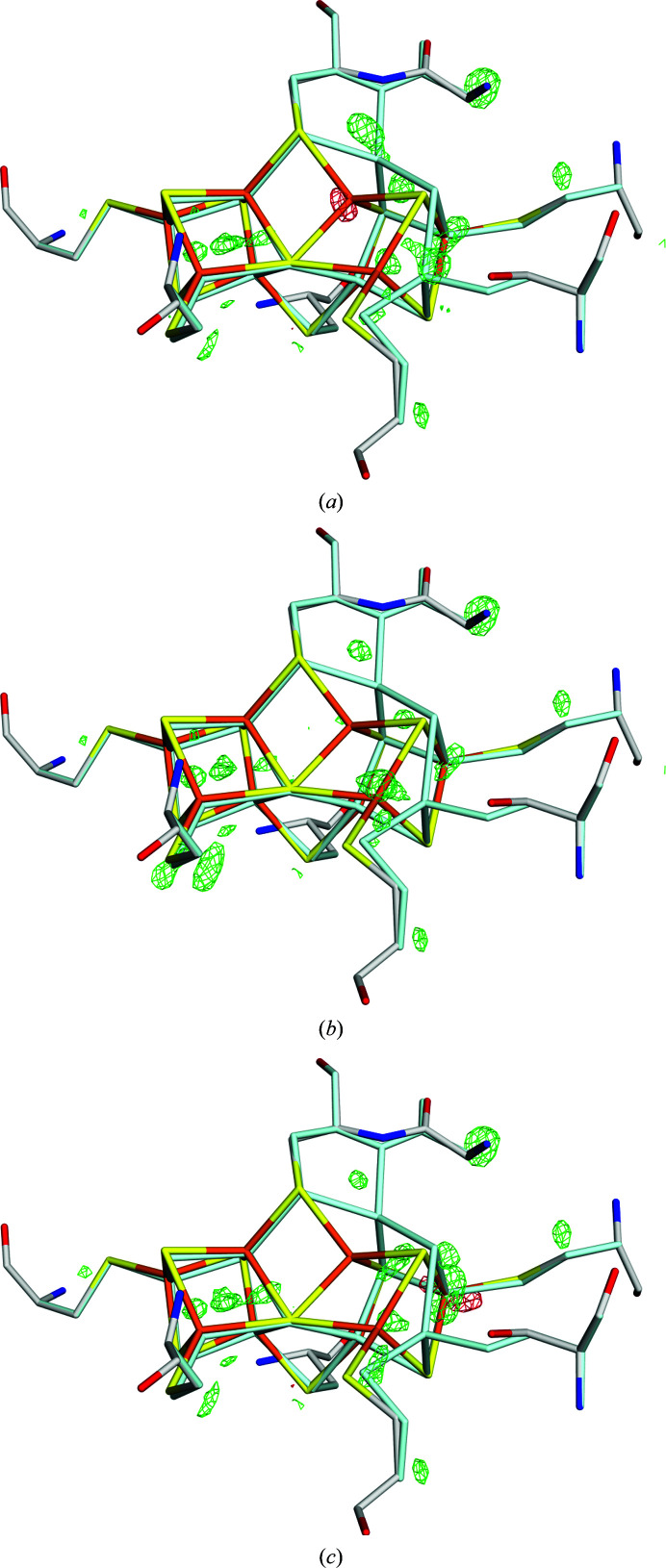
The *mF*_o_ − *DF*_c_ electron-density difference maps of the structure with PDB code 3u7q at the +4σ (green) and −4σ (red) levels for quantum-refined structures with *w*_A_ = 0.1 and occupancies of (*a*) 20/80%, (*b*) 10/90% and (*c*) 15/85%. The P^N^ state is shown in atomic colours, whereas the P^2+^ state is shown in pale cyan

This is also illustrated by the difference electron densities in Figs. 4 ▸(*a*) and 4 ▸(*b*). The 20/80% occupancy structure is clearly worse than the 10/90% occupancy structure around the Fe5 ion, indicating an occupancy that is too high for the P^N^ position (red density) and an occupancy that is too low for the P^2+^ position (green density), as in the 10/90% occupancy structure (the 30/70% occupancy structure is appreciably worse, as was indicated by the RSZD scores). However, the 10/90% occupancy structure has a much larger positive difference density around the SG atom of Cys154C than the 20/80% occupancy structure. Moreover, for the Fe6 ion the 20/80% occupancy structure has significant positive density around the P^2+^ position, whereas the 10/90% occupancy structure has a significant (and slightly larger) positive difference density around the P^N^ position.

Therefore, we also obtained a quantum-refined structure with 15% and 85% occupancies. It gave a clear improvement compared with the 10/90% occupancy structure around Cys154C and Fe6, and it keeps the improvement around Fe5 compared with the 20/80% occupancy structure. However, it shows a clear deterioration around Fe7. Still, we think this is our best structure, considering the RSZD scores of the cluster and all coordinating residues (both the maximum value and the sum of all values have a minimum for this structure, as can be seen in Supplementary Table S1).

It can be seen that the strain energy of the P^N^ state increases with occupancy, from 6 kJ mol^−1^ at 10% to 17 kJ mol^−1^ at 30%. The strain energy of the P^2+^ state also increases with occupancy, but the effect is much smaller: from 23.5 kJ mol^−1^ at 70% occupancy to 24.3 kJ mol^−1^ at 90% occupancy.

**Figure 5:**
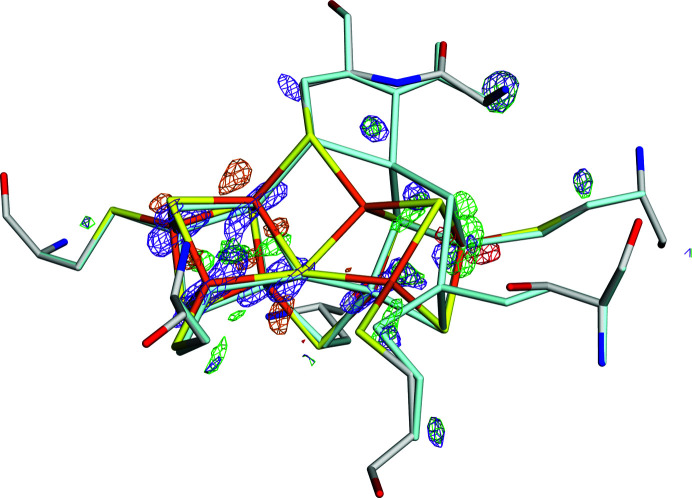
The *mF*_o_ − *DF*_c_ electron-density difference maps at a ±4σ level of the quantum-refined structures with *w*_A_ = 0.1 and occupancies 15/85% (green and red) compared with the original crystal structure of PDB entry 3u7q (purple and orange). The P^N^ state is shown in atomic colours, whereas the P^2+^ state is shown in pale cyan.

In Fig. 5 ▸ we compare the difference density maps of the best quantum-refined structure (*w*_A_ = 0.1 and 15/85% occupancy) and the original crystal structure. It can be seen that the quantum-refined structure describes the P-cluster much better than the original crystal structure. Interestingly, the improvement is largest for the Fe1–Fe4 subcluster. This can also be seen from the RSZD factors. The RSZD score for the P-cluster is 22 in the original crystal structure, but is only 8 in the quantum-refined structure. The *R*_free_ factor is similar for the two structures, as expected. However, the QM strain energies are very large for the crystal structure at 147 and 67 kJ mol^−1^, compared with 10 and 24 kJ mol^−1^ for the quantum-refined structure. This all clearly illustrates the advantage of our new *ComQumX*-2*QM* approach.

Finally, we also performed a coordinate refinement of the structure with PDB code 3u7q in which we used no restraints for the P-cluster (neither QM nor MM; *i.e.* no bonds were defined and the van der Waals parameters were zeroed). As in the original crystal structure, we included two conformations of Fe5 and Fe6 with occupancies of 0.8 and 0.2. The results in Table 1 ▸ shows that it gave *R* factors similar to those of the original crystal structure. However, the RZSD of the P-cluster is better at 14.2, but is worse than the best quantum-refined structures. The strain energies are high at 18 and 98 kJ mol^−1^.

### 3.3. PDB entry 6cdk   

Next, we performed a similar study based on the recent crystal structure of the putative P^1+^ state, PDB entry 6cdk (Keable *et al.*, 2018 ▸). It has an appreciably lower resolution, 2.10 Å, so it will illustrate the performance of our new *ComQumX*-2*QM* approach at a more modest resolution.

In the original crystal structure, only one set of coordinates were provided: those for the P^1+^ state. However, our QM/MM and quantum-refinement study of the structure indicated that it is rather a mixture of the P^1+^ and P^2+^ states (Cao *et al.*, 2019 ▸). Therefore, we ran a *ComQumX*-2*QM* refinement with the default *CNS* *w*_A_ weight (3.68) and equal occupancies (50%) of the P^1+^ and P^2+^ states. Using this structure, we then ran an occupancy refinement. Interestingly, it converged to average occupancies of 47% and 53% for the P^1+^ and P^2+^ states, respectively. Therefore, we kept the occupancies at 50/50%.

Next, we performed an investigation of the effect of the *w*_A_ weight in the same way as for PDB entry 3u7q. The results in Table 2 ▸ show similar trends as for the other crystal structure, although a bit more erratic. The *R* factor shows a minimum at *w*_A_ = 1, whereas the *R*_free_ factor shows an optimum at *w*_A_ = 10. The RSZD score is small for the P-cluster in all calculations, 1.4–2.5, and it is actually lowest for *w*_A_ = 0.3. This shows that all structures are in accordance with the crystallographic data, even those of the QM/MM calculation (*w*_A_ = 0, giving an RSZD of 2.5). The same also applies to the coordinating residues (Supplementary Table S2), giving maximum RSZD scores of 1.7–3.0, with a minimum for *w*_A_ = 1. In fact, the results show a significant variation with the details of the final *Phenix* *B*-factor refinement, for example the starting *B* factors.

**Figure 6:**
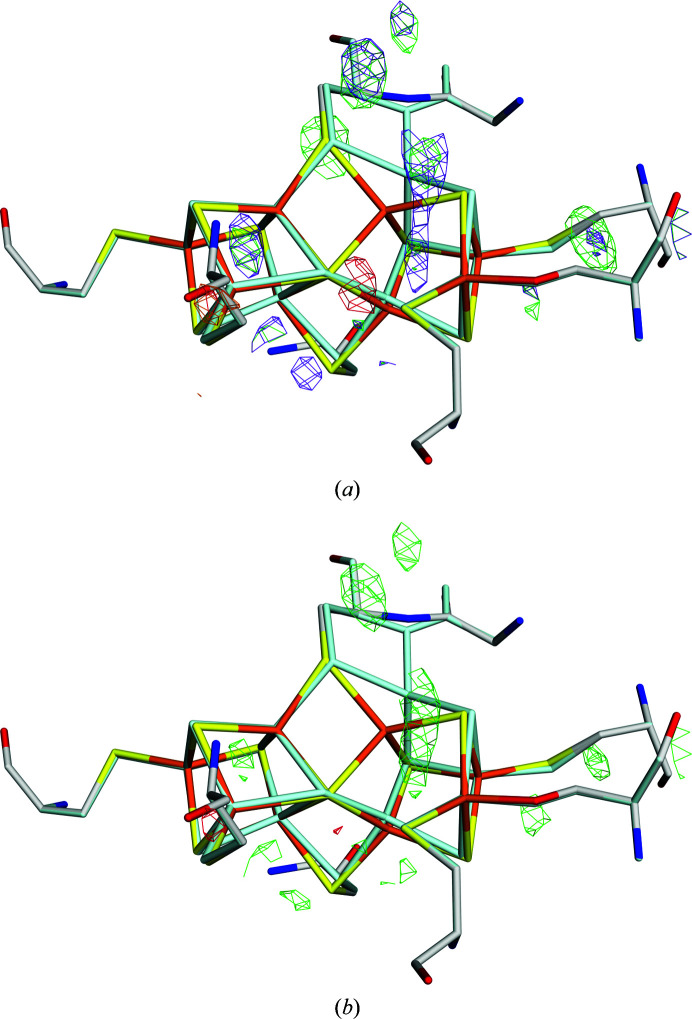
(*a*) The *mF*_o_ − *DF*_c_ electron-density difference maps of the quantum-refined structures of PDB entry 6cdk with (*a*) *w*_A_ = 1 (positive, green; negative, red) and *w*_A_ = 10 (positive, purple; negative, orange), as well as (*b*) the original crystal structure (positive, green; negative, red). All maps are contoured at the ±2.5σ level. The P^1+^ state is shown with atomic colours and the P^2+^ state is shown in pale cyan.

Supplementary Fig. S2 shows the 2*mF*_o_ − *DF*_c_ maps for the quantum-refined structures at *w*_A_ = 1. It can be seen that the electron density is much less well defined than for the structure with PDB code 3u7q. Fig. 6 ▸(*a*) compares the *mF*_o_ − *DF*_c_ difference maps for the quantum-refined structures at *w*_A_ = 1 and 10. Neither of the two structures shows any difference density at the 3σ level, so the maps are contoured at 2.5σ. The two structures are of comparable quality. The *w*_A_ = 1 structure has a negative density between the two clusters (red in Fig. 6 ▸ *a*) and a positive density around CysC88 (green), whereas the *w*_A_ = 10 structure has larger positive densities in the second cubane subcluster (violet).

The strain energies (Table 2 ▸) show the expected increase with *w*_A_. However, in contrast to the results in Table 1 ▸, the strain energies of the P^1+^ and P^2+^ states are similar except at the highest weights. This reflects that they have the same occupancy. Moreover, they are appreciably larger than for PDB entry 3u7q. This reflects the lower resolution of PDB entry 6cdk. Clearly, the strain energies of 93–109 kJ mol^−1^ for refinement at *w*_A_ = 10 are too large to be reasonable (they were 10 and 24 kJ mol^−1^ in the best structure for PDB entry 3u7q). Since the crystallographic criteria do not clearly point out any best structure, we tend to prefer the *w*_A_ = 1 structure, which gives more reasonable strain energies of 7–8 kJ mol^−1^.

For both *w*_A_ = 1 and 10, we repeated the occupancy refinement, which showed that the preferred occupancies are still close to equal: 44/56% and 48/52%, respectively. We also performed quantum refinement with 40/60% and 60/40% occupancies. The results in Table 2 ▸ show that the 50/50% occupancy gives the best results for most quality criteria, but not fully conclusively owing to the limited accuracy of the structure.

Fig. 6 ▸(*b*) shows the difference electron-density maps of the original crystal structure. It can be seen that the difference densities in the P-cluster are similar to (but are slightly larger than) those of the quantum-refined structure with *w*_A_ = 10. However, the RSZD score of the P-cluster is larger at 2.6 compared with 1.7 and 1.5 for the quantum-refined structures with *w*_A_ = 1 and 10, respectively. The effect is even larger for the coordinating residues (Supplementary Table S2), so that the sum of the RSZD scores of all coordinating residues is more than twice as large in the crystal structure than in the quantum-refined structures. Moreover, the strain energy of the P^1+^ state (which is the only state in the structure with PDB code 6cdk) is extremely high at 1406 kJ mol^−1^, illustrating that the structure is chemically totally unrealistic. This clearly shows the advantages of the quantum-refined structures.

We also performed a coordinate refinement without any empirical restraints at all (*i.e.* neither QM nor MM) for the P-cluster (P^1+^ state only). The results are also included in Table 2 ▸ (row No-MM). It can be seen that it gives *R* factors close to those of the original crystal structure, but the RSZD score of the P-cluster is twice as large at 5.4. The strain energy is also very large at 1160 kJ mol^−1^, showing that the structure is unrealistic.

**Figure 7:**
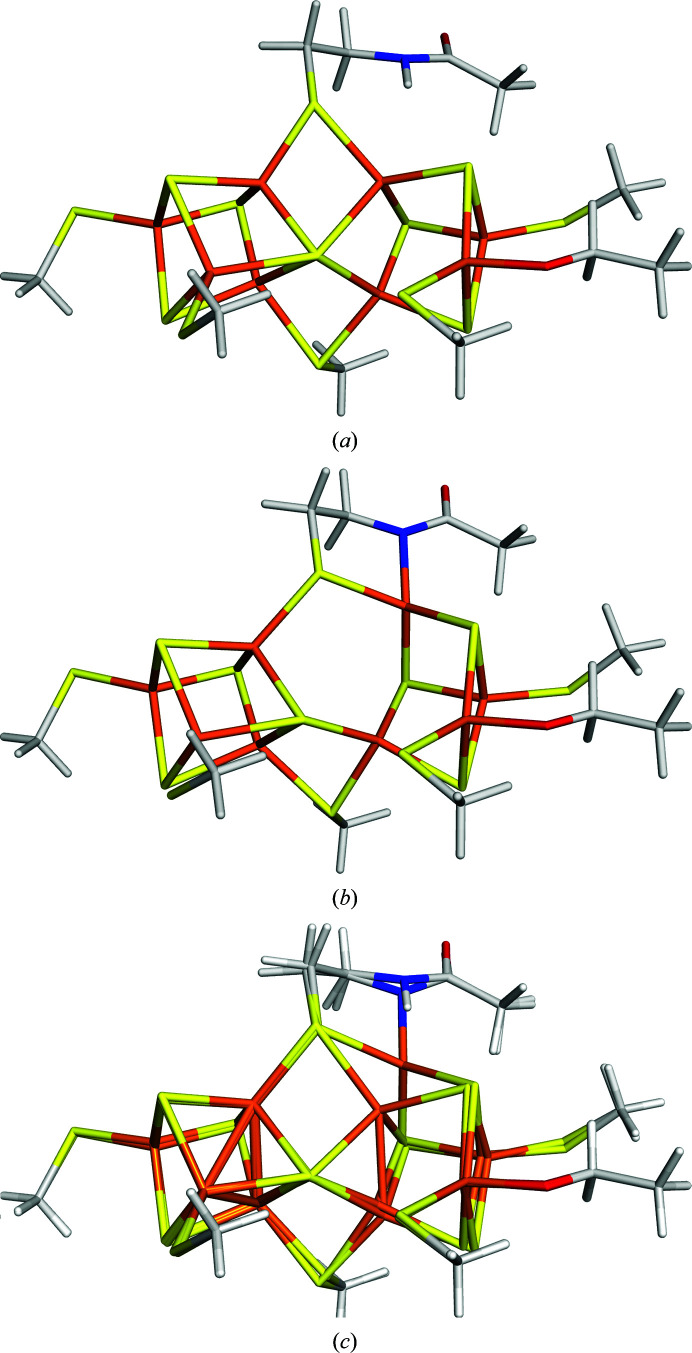
The *ComQumX*-2*QM* structure of PDB entry 6cdk for the (*a*) P^1+^ and (*b*) P^2+^ states with *w*_A_ = 1. (*c*) Overlay of the two structures.

The quantum-refined structures of the P^1+^ and P^2+^ states (both with 50% occupancy; *w*_A_ = 1) are shown in Fig. 7 ▸. In the P^1+^ state, the OG atom of SerD118 is deprotonated and coordinates to Fe6 with an Fe–O distance of 1.97 Å. On the other hand, the backbone N atom of CysC88 is protonated and does not coordinate to Fe5 (the Fe–N distance is 3.36 Å). This gives a distorted structure with one intact cubane (Fe1–Fe4), whereas the other cubane is missing one corner (the Fe6–S1 distance is 3.56 Å).

In the P^2+^ structure, both the OG atom of SerD118 and the N atom of CysC88 are deprotonated, with Fe–O and Fe–N distances of 1.92 and 2.04 Å, respectively. Thereby, the Fe5—S1 bond is also broken (3.78 Å distance). Compared with the P^1+^ structure, the Fe5 ion has moved by 1.29 Å. However, the Fe8 ion has moved by 0.34 Å and S2B by 0.27 Å, as can be seen in Fig. 7 ▸(*c*). The N atom of CysC88 has moved by 0.32 Å and the SG atom of CysD95 by 0.24 Å.

The quantum-refined structure of the P-cluster in the P^2+^ state is quite similar to that of the same state in the crystal structure with PDB code 3u7q. The MAD of the Fe–Fe distances is 0.07 Å, with a maximum deviation of 0.19 Å for the Fe6–Fe8 distance. For the Fe—S bonds, the MAD is 0.03 Å and the maximum deviation is 0.07 Å for the Fe8—S1 bond. Still, these are appreciably larger deviations than for the quantum-refined structure with PDB code 3u7q, showing that the lower resolution of PDB entry 6cdk still has a significant influence on the structure.

When the quantum-refined P^1+^ structure is compared with the original crystal structure with PDB code 6cdk, the deviations are even larger, with MADs of 0.18 and 0.26 Å for the Fe–Fe distances and Fe—S bonds, respectively, and with maximum deviations of 0.41 Å for the Fe1–Fe2 distance and 0.84 Å for the Fe1—S3A bond. The reason for this is that the original structure with PDB code 6cdk involves a mixture of the P^1+^ and P^2+^ states, giving coordinates that are weighted averages of the two states, which therefore are not correct for either of the two states. Moreover, the structure involves some strongly dubious distances, as has been discussed previously (Cao *et al.*, 2019 ▸). Therefore, the current quantum-refined structures are much more reliable and should represent the most reliable structures of the P^1+^ state of the P-cluster.

Compared with our previous quantum-refined structure of the P^1+^ state in the crystal structure with PDB code 6cdk (without dual conformations, *i.e.* with the P-cluster modelled with 100% of the P^1+^ state; Cao *et al.*, 2019 ▸), the MADs of the Fe–Fe distances and Fe—S bond lengths are 0.14 and 0.08 Å, respectively. The maximum deviations are 0.44 Å for the Fe5–Fe6 distance and 0.95 Å for the Fe5—S1 bond length. Both of these largest deviations are caused by the fact that Fe5 ends up in a position that is not ideal for the P^1+^ state, but instead is a compromise between the P^1+^ and P^2+^ states. This problem is solved with the current *ComQumX*-2*QM* approach.

The new quantum-refined structures are much closer to our previous QM/MM structures (Cao *et al.*, 2019 ▸), even if they were based on the crystal structure with PDB code 3u7q. The MADs of the Fe–Fe distances and Fe—S bonds are both 0.06 Å for the P^1+^ state, with maximum deviations of 0.14 and 0.23 Å for the Fe2–Fe4 distance and the Fe6—S1 bond length, respectively. For the P^2+^ state, the corresponding MADs are 0.05 and 0.02 Å, with maximum deviations of 0.20 Å for the Fe4–Fe5 distance and 0.05 Å for the Fe5—SG bond length. This shows that the quantum-refined structures are quite similar to the QM/MM structures, but still contain some information from the crystal structure with PDB code 6cdk, as expected.

## 4. Conclusions   

In this study, we have extended the quantum-refinement approach to allow dual conformations within the QM system, *i.e.* within the system of central interest. Computationally, it is a rather simple extension, simply doubling the calculations of the QM system (both at the QM and MM level) and avoiding double counting of any interaction or energy term. As for our standard quantum-refinement software, *ComQum-X*, it is implemented as a combination of the QM program package *TURBOMOLE* and the crystallography software *CNS*. The interface is available from the authors upon request. The approach can trivially be extended to more conformations. For example, we have already performed an application to a guanidinium quadruplex with an Au^3+^ inhibitor showing four distinct conformations (to be published). Naturally, the approach takes approximately twice as much computational resources than standard quantum refinement, which itself is appreciably more demanding than standard crystallographic refinement. Still, the refinements in this study (employing a QM system of 62–64 atoms) typically take less than one day on a single processor.

In practice, this approach may be used to solve important problems in crystallography and biochemistry, as the two applications to the P-cluster in nitrogenase show. Even at a high resolution, it is not possible to discern distinct positions of atoms with dual conformations if they are less than ∼1 Å apart. This is illustrated by the structure with PDB code 3u7q (at 1.0 Å) of the P-cluster with a 20/80% mixture of the P^N^ and P^2+^ states. In the original structure, only two atoms, Fe5 and Fe6, have different coordinates, being 1.3–1.4 Å apart. The reason for this is that the ideal structures of the P^N^ and P^2+^ states are not available in the empirical (*i.e.* MM) restraints used in standard refinement. However, the QM calculations can automatically provide such restraints, thereby providing reliable structures of the two oxidation states inside the crystal structure. They show that also some of the other atoms move by up to 0.7 Å. Most importantly, the RSZD scores and the difference electron-density maps show that the quantum-refined structure provides an improved description of the P-cluster.

The structure with PDB code 6cdk, at lower resolution (2.1 Å), was originally suggested to only contain the elusive P^1+^ state (Keable *et al.*, 2018 ▸). However, our previous QM/MM and quantum-refinement study has already shown that it is actually a mixture of the P^1+^ and P^2+^ states, which therefore suggests some strongly dubious Fe–Fe and Fe–S distances (Cao *et al.*, 2019 ▸). However, quantum refinement with only a single QM system could not provide any reliable structures of either of the two states, because the experimental data present a mixture of both. This dilemma is solved by the present *ComQumX*-2*QM* approach, which gives chemically reasonable structures of both states which are in accordance with the crystallographic raw data. The strain energy is much lower for the quantum-refined structures, showing that they make more chemical sense, and the RSZD scores are slightly lower than for the original crystal structure.

Thus, our new *ComQumX*-2*QM* approach provides a novel tool in crystallography and computational chemistry to interpret complicated crystal structures. It is particularly useful for parts of the structure that contain nonstandard residues (heterocompounds), *i.e.* substrates, inhibitors or metal sites, for which no accurate empirical restraints are available. Such compounds are typically found in the active sites, *i.e.* the mechanistically most interesting parts of the proteins. In particular, it will be important for metal sites, which often are partly reduced during data collection. In future studies, we will continue to apply this approach to other interesting sites in proteins.

## Supplementary Material

Supplementary Figures and Tables. DOI: 10.1107/S2059798320012917/ag5040sup1.pdf

## Figures and Tables

**Table 1:** Results of quantum refinements of the crystal structure with PDB code 3u7q with different values of the weight factor *w*_A_, showing the *R*_free_ and *R*_work_ factors and the RSZD score of the P-cluster (the maximum value for the Fe and S atoms in the P-cluster in the two conformations), as well as the strain energy of the QM system The first line shows the results for the original crystal structure and the second line shows the results for a refinement in which no QM or MM data are used for the P-cluster.

	Occupancies					Strain energy (kJ mol^−1^)	
w_A_	P^N^	P^2+^	*R*_free_	*R*_work_	RSZD	P^N^	P^2+^
3u7q	0.20	0.80	0.1602	0.1439	22.1	147.0	66.8
No-MM	0.20	0.80	0.1601	0.1433	14.2	18.4	98.4
0.00	0.20	0.80	0.1642	0.1480	99.9	0.0	0.0
0.01	0.20	0.80	0.1604	0.1442	21.4	1.1	10.4
0.03	0.20	0.80	0.1603	0.1440	13.6	4.3	17.5
0.08	0.20	0.80	0.1602	0.1438	10.4	8.6	22.9
0.10	0.20	0.80	0.1601	0.1437	9.0	10.2	23.7
0.30	0.20	0.80	0.1601	0.1438	9.1	31.3	24.6
0.10	0.10	0.90	0.1602	0.1438	5.1	5.8	24.3
0.10	0.15	0.85	0.1602	0.1439	7.7	8.1	24.0
0.10	0.20	0.80	0.1601	0.1437	9.0	10.2	23.7
0.10	0.30	0.70	0.1600	0.1437	24.6	16.6	23.5

**Table 2:** Results of quantum refinements of the crystal structure with PDB code 6cdk with different values of the weight factor *w*_A_ and the occupancies, showing the *R*_free_ and *R*_work_ factors and the RSZD score of the P-cluster (the maximum value for the Fe and S atoms in the P-cluster in the two conformations), as well as the strain energy of the QM system The first line shows the results for the original crystal structure and the second line shows the results for a refinement in which no QM or MM data are used for the P-cluster.

	Occupancies					Strain energy (kJ mol^−1^)	
*w*_A_	P^1+^	P^2+^	*R*_free_	*R*_work_	RSZD	P^1+^	P^2+^
6cdk	1.0	0.0	0.2581	0.2118	2.6	1406.4	
No-MM	1.0	0.0	0.2577	0.2116	5.4	1159.9	
0.0	0.5	0.5	0.2588	0.2049	2.5	0.0	0.0
0.1	0.5	0.5	0.2585	0.2049	2.2	0.2	0.2
0.3	0.5	0.5	0.2588	0.2049	1.4	1.5	1.2
1.0	0.5	0.5	0.2583	0.2038	1.7	7.6	6.8
3.7	0.5	0.5	0.2586	0.2046	1.6	39.1	32.5
10.0	0.5	0.5	0.2577	0.2050	1.5	109.4	92.6
30.0	0.5	0.5	0.2579	0.2051	2.5	222.0	262.7
100.0	0.5	0.5	0.2578	0.2058	1.8	542.0	667.0
1.0	0.4	0.6	0.2584	0.2043	1.5	3.8	9.5
1.0	0.5	0.5	0.2583	0.2038	1.7	7.6	6.8
1.0	0.6	0.4	0.2589	0.2044	2.5	11.8	4.3
10.0	0.4	0.6	0.2578	0.2046	2.0	82.9	103.7
10.0	0.5	0.5	0.2577	0.2050	1.5	109.4	92.6
10.0	0.6	0.4	0.2578	0.2052	2.4	131.7	81.4
