# Supplementary material for: Quantum refinement with multiple conformations: application to the P-cluster in nitrogenase
Source: Acta Crystallogr D Struct Biol. 2020 Oct 16;76(Pt 11):1145–56. doi: 10.1107/S2059798320012917 (PMC7604908; doi:10.1107/S2059798320012917)
Supplement: Supplementary file 1 [file d-76-01145-sup1.pdf]

STRUCTURAL
BIOLOGY

Volume 76 (2020)

Supporting information for article:

Quantum refinement with multiple conformations: application to the P-cluster in nitrogenase

Lili Cao and Ulf Ryde

Table S1. RSZD scores of residues in the QM system of the quantum refinements of the 3U7Q crystal structure with different values of the w_A weight factor. The residues are Cys-C62, Gly-C87, Cys-C88, Cys-C154, Cys-D70, Cys-D95, Cys-D153 and Ser-D188. Max and Sum in the last two columns are the maximum and the sum of all the 18 RSZD scores in the table for each structure.

w_A	occupancy		P^N									P^{2+}									Max	Sum
	P^N	P^{2+}	C62	G87	C88	C154	C70	C95	C153	S88	FeS	C62	G87	C88	C154	C70	C95	C153	S88	FeS		
3U7Q	0.20	0.80	1.5	0.0	0.5	0.0	0.0	0.8	1.1	0.0	1.8	0.4	4.0	2.1	2.0	1.9	0.1	4.6	0.8	22.1	22.1	43.7
no-MM	0.20	0.80	1.5	0.0	0.3	0.0	0.3	0.7	1.3	0.0	3.1	0.3	4.4	0.4	2.1	2.2	0.0	4.5	0.7	14.2	14.2	36.0
0.00	0.20	0.80	8.5	9.9	0.4	6.9	5.9	1.7	6.9	4.3	28.8	27.1	18.9	9.4	30.3	27.8	5.1	99.9	4.7	99.9	99.9	396.4
0.01	0.20	0.80	1.6	1.0	0.5	0.4	3.6	0.7	5.5	0.3	7.1	3.8	12.4	3.5	5.3	7.8	0.9	18.7	1.3	21.4	21.4	95.8
0.03	0.20	0.80	1.3	0.6	0.3	0.0	1.1	0.7	4.2	0.0	5.3	1.2	7.0	0.5	2.6	3.2	0.1	8.9	0.9	13.6	13.6	51.5
0.08	0.20	0.80	1.4	0.0	0.5	0.0	0.0	0.7	1.6	0.0	4.4	0.3	4.8	0.3	2.2	2.2	0.0	5.9	0.6	10.4	10.4	35.3
0.10	0.20	0.80	1.5	0.0	0.5	0.0	0.0	0.7	1.4	0.0	4.7	0.2	4.6	0.4	2.3	2.2	0.0	4.8	0.6	9.0	9.0	32.9
0.30	0.20	0.80	1.5	0.0	0.4	0.0	0.0	0.7	1.1	0.0	4.4	0.1	3.7	0.4	1.9	1.9	0.0	3.2	0.3	9.1	9.1	28.7
0.10	0.10	0.90	1.5	0.0	0.3	0.3	0.1	0.6	1.5	0.0	5.1	0.7	5.1	0.5	9.1	3.5	0.2	5.8	0.9	3.8	9.1	39.0
0.10	0.15	0.85	1.7	0.0	0.5	0.0	0.0	0.6	1.1	0.0	0.9	0.4	4.9	0.6	3.2	2.8	0.1	6.7	0.8	7.7	7.7	32.0
0.10	0.20	0.80	1.5	0.0	0.5	0.0	0.0	0.7	1.4	0.0	4.7	0.2	4.6	0.4	2.3	2.2	0.0	4.8	0.6	9.0	9.0	32.9
0.10	0.30	0.70	1.6	0.0	0.4	0.0	0.0	0.8	1.1	0.0	17.3	0.1	3.9	0.4	1.2	1.5	0.0	4.2	0.3	24.6	24.6	57.4

Table S2. RSZD scores of residues in the QM system of the quantum refinements of the 6CDK crystal structure with different values of the w_A weight factor. The residues are Cys-C62, Gly-C87, Cys-C88, Cys-C154, Cys-D70, Cys-D95, Cys-D153 and Ser-D188. Max and Sum in the last two columns are the maximum and the sum of all the 18 RSZD scores in the table for each structure.

w_A	occupancy		P^{1+}									P^{2+}									Max	Sum
	P^{1+}	P^{2+}	C62	G87	C88	C154	C70	C95	C153	S88	FeS	C62	G87	C88	C154	C70	C95	C153	S88	FeS		
6CDK	1.0	0.0	0.2	0.3	1.9	1.2	1.5	1.0	1.7	1.3	2.6										2.6	23.4
no-MM	1.0	0.0	2.1	0.6	2.7	1.0	1.3	6.5	0.2	3.0	5.4										6.5	45.6
0.0	0.2	0.8	1.1	0.1	1.0	1.7	0.8	0.2	1.3	0.5	2.5	0.4	0.3	1.1	0.1	0.3	0.1	1.8	0.1	1.6	2.5	15.0
0.1	0.2	0.8	1.3	0.0	1.1	1.8	0.8	0.3	2.8	0.4	2.2	0.0	0.3	0.9	0.2	0.5	0.1	1.7	0.0	1.1	2.8	15.5
0.3	0.2	0.8	1.1	0.0	1.4	1.8	1.5	0.1	3.0	0.3	1.1	0.0	0.1	1.2	0.2	0.4	0.1	1.0	0.0	1.4	3.0	14.7
1.0	0.2	0.8	1.0	0.3	0.6	0.7	0.0	0.1	1.2	0.2	1.7	0.1	0.2	0.9	0.0	0.9	0.5	0.4	0.0	1.4	1.7	10.2
3.7	0.2	0.8	1.1	0.0	0.2	1.8	0.1	0.2	0.3	0.6	1.5	0.0	0.0	0.9	0.2	0.5	0.4	0.1	0.0	1.6	1.8	9.5
10.0	0.2	0.8	1.3	0.0	1.0	1.9	0.3	0.2	1.3	0.9	1.1	0.0	0.0	0.1	0.4	0.2	0.9	0.0	0.0	1.5	1.9	11.1
30.0	0.2	0.8	1.0	0.0	0.7	2.0	0.3	0.4	1.3	0.8	0.9	0.0	0.0	0.0	0.4	1.3	0.6	0.0	0.0	2.5	2.5	12.2
100.0	0.2	0.8	0.9	0.0	0.4	1.9	0.1	0.7	0.8	0.4	0.4	0.0	0.0	0.2	0.4	0.2	0.9	0.1	0.0	1.8	1.9	9.2
1.0	0.4	0.6	1.1	0.0	0.9	2.2	0.0	0.2	2.2	0.3	0.2	0.0	0.1	1.1	0.4	1.0	0.3	0.3	0.0	1.5	2.2	11.8
1.0	0.2	0.8	1.0	0.3	0.6	0.7	0.0	0.1	1.2	0.2	1.7	0.1	0.2	0.9	0.0	0.9	0.5	0.4	0.0	1.4	1.7	10.2
1.0	0.6	0.4	1.1	0.1	1.5	1.8	0.1	0.1	0.3	0.3	2.5	0.0	0.1	1.3	0.1	0.6	0.0	0.1	0.0	1.3	2.5	11.3
10.0	0.4	0.6	1.3	0.0	0.8	1.9	0.3	0.3	1.1	0.5	2.0	0.0	0.0	0.3	0.7	0.9	0.6	0.1	0.0	1.1	2.0	11.9
10.0	0.2	0.8	1.3	0.0	1.0	1.9	0.3	0.2	1.3	0.9	1.1	0.0	0.0	0.1	0.4	0.2	0.9	0.0	0.0	1.5	1.9	11.1
10.0	0.6	0.4	1.2	0.0	1.0	1.9	0.3	0.2	1.2	0.6	1.2	0.0	0.0	0.0	0.3	0.3	0.9	0.0	0.0	2.4	2.4	11.5

Figure S1. The $2mF_o - DF_c$ electron-density map at a 6σ level of the quantum-refined structure with $w_A = 0.1$ of the 3U7Q structure. The P^N state is shown in atomic colours, whereas the P^{2+} state is show in cyan.

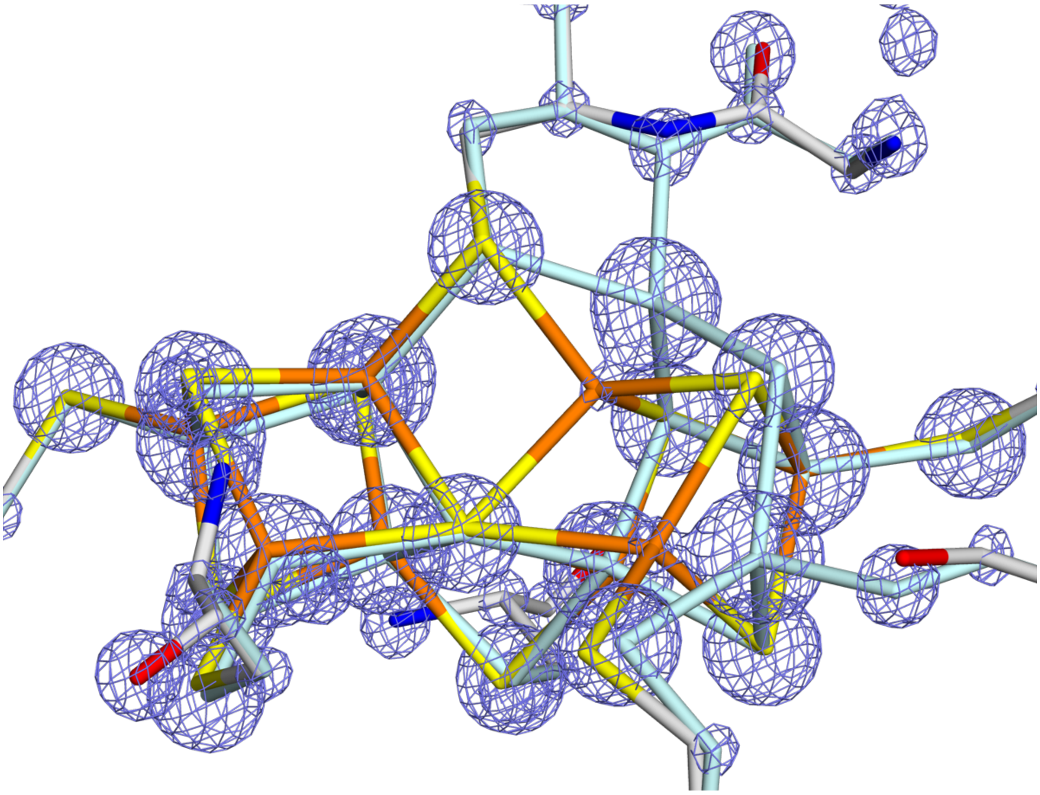

Figure S2. The $2mF_o - DF_c$ electron-density map at a 4 σ level of the quantum-refined structure with $w_A = 1$ of the 6CDK structure. The P^{1+} state is shown in atomic colours, whereas the P^{2+} state is show in pale cyan.

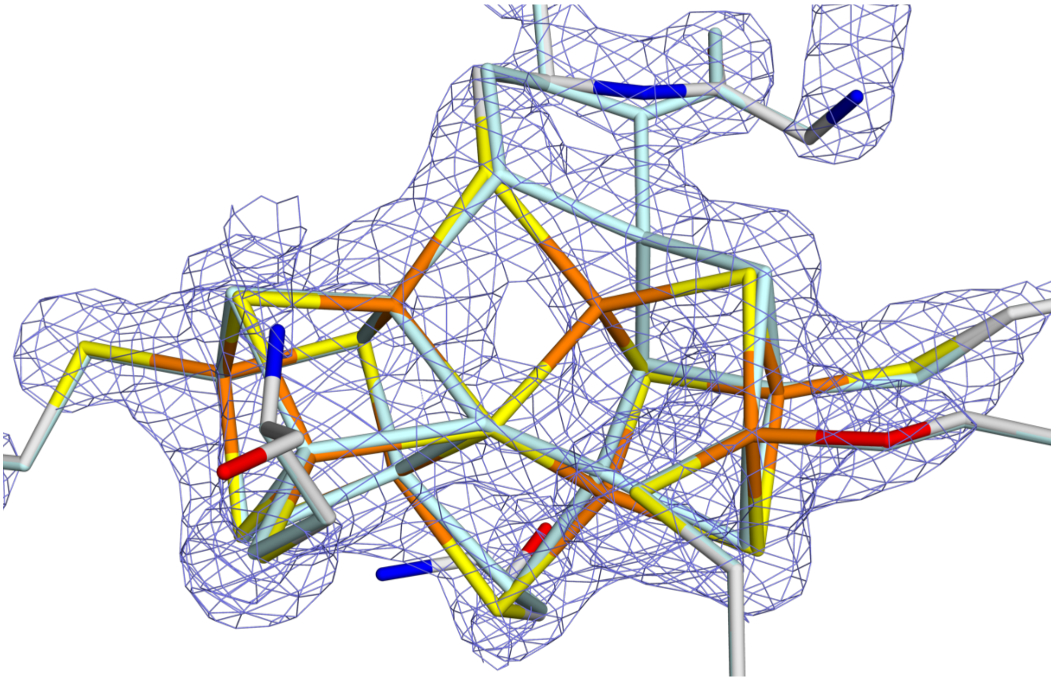

Table S3. Final quantum-refined coordinates of the P-cluster in the (a) 3U7Q and (b) 6CDK structures (w_A = 0.1 and 1, with 15/85% and 50/50% occupancy, respectively).

(a)

```
REMARK Quantum-refined structure of 3U7Q, obtained with wA=0.1 and 15/85 occupancy
ATOM   8427  CA BCYS C  62      44.704  -3.374 -10.608  0.20  8.00      CAC1 C
ANISOU 8427  CA BCYS C  62     1059    963   1016     19    122    -59  CAC1 C
ATOM   8428  CB BCYS C  62      45.059  -4.825 -10.169  0.20  7.94      CAC1 C
ANISOU 8428  CB BCYS C  62     1024    989   1006     40     40    -90  CAC1 C
ATOM   8429  SG BCYS C  62      43.648  -5.510  -9.161  0.20  7.57      CAC1 S
ANISOU 8429  SG BCYS C  62      895    997    983     25   -152    -93  CAC1 S
ATOM   8607  N  BGLY C  87      41.738 -13.653  -1.289  0.20  8.90      CAC1 N
ANISOU 8607  N  BGLY C  87     1186   1094   1102     79     23    -61  CAC1 N
ATOM   8608  CA BGLY C  87      42.151 -12.280  -1.050  0.20  9.39      CAC1 C
ANISOU 8608  CA BGLY C  87     1248   1168   1152     91      7    -20  CAC1 C
ATOM   8609  C  BGLY C  87      41.260 -11.202  -1.624  0.20  8.95      CAC1 C
ANISOU 8609  C  BGLY C  87     1120   1154   1127    113      2    -48  CAC1 C
ATOM   8610  O  BGLY C  87      40.617 -10.406  -0.918  0.20  9.53      CAC1 O
ANISOU 8610  O  BGLY C  87     1223   1191   1206    189     56   -105  CAC1 O
ATOM   8612  N  BCYS C  88      41.264 -11.144  -2.985  0.20  8.36      CAC1 N
ANISOU 8612  N  BCYS C  88      987   1109   1081    102    -25    -21  CAC1 N
ATOM   8613  CA BCYS C  88      40.865  -9.922  -3.658  0.20  8.22      CAC1 C
ANISOU 8613  CA BCYS C  88      932   1099   1092     72    -34     -9  CAC1 C
ATOM   8614  C  BCYS C  88      39.426  -9.594  -3.259  0.20  7.90      CAC1 C
ANISOU 8614  C  BCYS C  88      935   1011   1056      9     -2    -37  CAC1 C
ATOM   8615  CB BCYS C  88      40.891 -10.049  -5.198  0.20  8.29      CAC1 C
ANISOU 8615  CB BCYS C  88      875   1181   1093     59    -83    -31  CAC1 C
ATOM   8616  SG BCYS C  88      42.083 -11.271  -5.905  0.20  7.85      CAC1 S
ANISOU 8616  SG BCYS C  88      727   1251   1002     70   -304     25  CAC1 S
ATOM   9132  CA BCYS C 154      44.943 -13.257 -11.480  0.20  8.26      CAC1 C
ANISOU 9132  CA BCYS C 154     1001   1088   1049    138    117    -51  CAC1 C
ATOM   9133  CB BCYS C 154      46.223 -12.871 -10.698  0.20  8.55      CAC1 C
ANISOU 9133  CB BCYS C 154      954   1161   1132    157    150    -67  CAC1 C
ATOM   9134  SG BCYS C 154      46.586 -11.080 -10.645  0.20  8.05      CAC1 S
ANISOU 9134  SG BCYS C 154      808   1200   1051    170     26    -85  CAC1 S
ATOM  12331  CA BCYS D  70      45.148 -14.550   1.896  0.20  7.46      DAC1 C
ANISOU12331  CA BCYS D  70      878   1028    929     99     21   -104  DAC1 C
ATOM  12332  CB BCYS D  70      45.149 -14.244   0.348  0.20  7.39      DAC1 C
ANISOU12332  CB BCYS D  70      795   1073    939     84    -89    -71  DAC1 C
ATOM  12333  SG BCYS D  70      45.768 -12.617  -0.227  0.20  6.65      DAC1 S
ANISOU12333  SG BCYS D  70      518   1104    904     50   -373    -17  DAC1 S
ATOM  12511  CA BCYS D  95      47.412  -6.954  -4.077  0.20  7.72      DAC1 C
ANISOU12511  CA BCYS D  95      945   1001    988     97     25    -55  DAC1 C
ATOM  12512  CB BCYS D  95      48.260  -8.224  -4.099  0.20  7.86      DAC1 C
ANISOU12512  CB BCYS D  95      900   1015   1072    131    -32    -99  DAC1 C
ATOM  12513  SG BCYS D  95      47.970  -9.278  -5.579  0.20  7.45      DAC1 S
ANISOU12513  SG BCYS D  95      708   1026   1097     68   -214   -138  DAC1 S
ATOM  12964  CA BCYS D 153      49.349 -15.574  -6.752  0.20  8.85      DAC1 C
ANISOU12964  CA BCYS D 153     1110   1178   1076    143     78    -86  DAC1 C
ATOM  12965  CB BCYS D 153      47.803 -15.791  -6.677  0.20  9.53      DAC1 C
ANISOU12965  CB BCYS D 153     1151   1318   1151     80     72   -117  DAC1 C
ATOM  12966  SG BCYS D 153      46.743 -14.388  -7.149  0.20  9.54      DAC1 S
ANISOU12966  SG BCYS D 153     1114   1383   1128    127     30   -104  DAC1 S
ATOM  13233  N  BSER D 188      48.423 -17.569  -2.220  0.20  9.08      DAC1 N
ANISOU13233  N  BSER D 188     1181   1040   1228    117    -29      3  DAC1 N
ATOM  13234  CA BSER D 188      47.073 -17.221  -1.732  0.20  9.36      DAC1 C
ANISOU13234  CA BSER D 188     1248   1051   1256     93     -2    -26  DAC1 C
ATOM  13235  C  BSER D 188      46.052 -18.362  -1.781  0.20  9.08      DAC1 C
ANISOU13235  C  BSER D 188     1226   1029   1194    110     50    -43  DAC1 C
ATOM  13236  CB BSER D 188      46.512 -16.014  -2.490  0.20 10.01      DAC1 C
ANISOU13236  CB BSER D 188     1366   1077   1361     33    -14     -3  DAC1 C
ATOM  13237  OG BSER D 188      46.209 -16.402  -3.822  0.20 10.16      DAC1 O
ANISOU13237  OG BSER D 188     1391   1082   1387    -44     29    -57  DAC1 O
ATOM  16041  S1 B1CL D7498      45.513 -11.344  -6.838  0.20  8.10      DAC1 S2-
ANISOU16041  S1 B1CL D7498      823   1182   1072     90   -280    -17  DAC1 S2-
ATOM  16042  S2AB1CL D7498      46.420  -7.963  -8.779  0.20  8.37      DAC1 S2-
ANISOU16042  S2AB1CL D7498      876   1248   1055     98   -114    -98  DAC1 S2-
ATOM  16043  S2BB1CL D7498      44.168 -13.721  -3.848  0.20  7.51      DAC1 S2-
```

```
ANISOU16043  S2BB1CL D7498       488   1260   1104     16   -184    -32  DAC1 S2-
ATOM  16044  S3AB1CL D7498       43.142  -9.562  -9.350  0.20  9.27      DAC1 S2-
ANISOU16044  S3AB1CL D7498      1276   1186   1060     24    195   -111  DAC1 S2-
ATOM  16045  S3BB1CL D7498       47.660 -12.747  -3.683  0.20  6.91      DAC1 S2-
ANISOU16045  S3BB1CL D7498       378   1153   1096    148   -208     38  DAC1 S2-
ATOM  16046  S4AB1CL D7498       43.936  -7.843  -6.150  0.20  7.96      DAC1 S2-
ANISOU16046  S4AB1CL D7498       764   1215   1046    217   -373    -10  DAC1 S2-
ATOM  16047  S4BB1CL D7498       44.992 -10.263  -3.075  0.20  7.17      DAC1 S2-
ANISOU16047  S4BB1CL D7498       556   1164   1004     26   -410     17  DAC1 S2-
ATOM  16048 FE1 B1CL D7498       44.121  -7.622  -8.470  0.20  9.01      DAC1Fe2+
ANISOU16048 FE1 B1CL D7498      1132   1180   1113    -73     58   -122  DAC1Fe2+
ATOM  16049 FE2 B1CL D7498       43.605  -9.910  -7.110  0.20  8.21      DAC1Fe2+
ANISOU16049 FE2 B1CL D7498       799   1266   1055    184   -476     15  DAC1Fe2+
ATOM  16050 FE3 B1CL D7498       45.403 -10.054  -8.943  0.20  8.28      DAC1Fe2+
ANISOU16050 FE3 B1CL D7498       814   1259   1073    169    -17   -135  DAC1Fe2+
ATOM  16051 FE4 B1CL D7498       45.989 -13.153  -5.292  0.20  9.43      DAC1Fe2+
ANISOU16051 FE4 B1CL D7498      1228   1180   1177    128    -66      7  DAC1Fe2+
ATOM  16052 FE5 B1CL D7498       44.194 -11.557  -4.806  0.20  9.54      DAC1Fe2+
ANISOU16052 FE5 B1CL D7498      1031   1469   1125     87   -151     32  DAC1Fe2+
ATOM  16053 FE6 B1CL D7498       45.889  -8.936  -6.747  0.20  7.21      DAC1Fe2+
ANISOU16053 FE6 B1CL D7498       430   1198   1111    132   -284     43  DAC1Fe2+
ATOM  16054 FE7 B1CL D7498       45.671 -12.419  -2.544  0.20  6.12      DAC1Fe2+
ANISOU16054 FE7 B1CL D7498       383   1059    884      1   -262    -49  DAC1Fe2+
ATOM  16055 FE8 B1CL D7498       46.599 -10.917  -4.625  0.20  7.15      DAC1Fe2+
ANISOU16055 FE8 B1CL D7498       517   1130   1068    -80   -251     81  DAC1Fe2+
ATOM  18772  CA ACYS C  62       44.703  -3.379 -10.604  0.80  8.46      CAC2 C
ANISOU18772  CA ACYS C  62      1154    983   1078    -30    199    123  CAC2 C
ATOM  18773  CB ACYS C  62       45.049  -4.833 -10.154  0.80  8.56      CAC2 C
ANISOU18773  CB ACYS C  62      1164   1005   1083     67    242     37  CAC2 C
ATOM  18774  SG ACYS C  62       43.653  -5.519  -9.139  0.80  7.94      CAC2 S
ANISOU18774  SG ACYS C  62      1038    947   1032     69     97     84  CAC2 S
ATOM  18781  N  AGLY C  87       41.729 -13.618  -1.274  0.80  7.93      CAC2 N
ANISOU18781  N  AGLY C  87      1142    860   1010    -40    203    -24  CAC2 N
ATOM  18782  CA AGLY C  87       42.091 -12.216  -0.982  0.80  8.23      CAC2 C
ANISOU18782  CA AGLY C  87      1126    965   1035     38     49    -57  CAC2 C
ATOM  18783  C  AGLY C  87       41.315 -11.091  -1.666  0.80  7.00      CAC2 C
ANISOU18783  C  AGLY C  87       816    885    957    -33     -6    -76  CAC2 C
ATOM  18784  O  AGLY C  87       40.624 -10.289  -0.986  0.80  7.48      CAC2 O
ANISOU18784  O  AGLY C  87       979    814   1049    -39    112   -178  CAC2 O
ATOM  18785  N  ACYS C  88       41.519 -11.031  -2.997  0.80  7.14      CAC2 N
ANISOU18785  N  ACYS C  88      1032    841    838    110    181    -76  CAC2 N
ATOM  18786  CA ACYS C  88       40.884  -9.924  -3.691  0.80  7.70      CAC2 C
ANISOU18786  CA ACYS C  88      1049    908    970    243     76     57  CAC2 C
ATOM  18787  C  ACYS C  88       39.426  -9.595  -3.264  0.80  7.45      CAC2 C
ANISOU18787  C  ACYS C  88      1008    874    947    130     27    -69  CAC2 C
ATOM  18788  CB ACYS C  88       40.795 -10.177  -5.205  0.80  8.44      CAC2 C
ANISOU18788  CB ACYS C  88      1181    945   1081    190    282     21  CAC2 C
ATOM  18789  SG ACYS C  88       42.216 -11.170  -5.830  0.80  7.44      CAC2 S
ANISOU18789  SG ACYS C  88      1039    807    980     57    197    -27  CAC2 S
ATOM  18790  CA ACYS C 154       44.951 -13.254 -11.467  0.80  8.81      CAC2 C
ANISOU18790  CA ACYS C 154      1227   1073   1047    132    -30    -67  CAC2 C
ATOM  18791  CB ACYS C 154       46.238 -12.891 -10.638  0.80  9.54      CAC2 C
ANISOU18791  CB ACYS C 154      1312   1105   1209    211     91     28  CAC2 C
ATOM  18792  SG ACYS C 154       46.732 -11.126 -10.560  0.80  8.53      CAC2 S
ANISOU18792  SG ACYS C 154      1181   1011   1047    237    147     24  CAC2 S
ATOM  18816  CA ACYS D  70       45.143 -14.558   1.901  0.80  7.72      DAC2 C
ANISOU18816  CA ACYS D  70       995    966    974    152     42    -63  DAC2 C
ATOM  18817  CB ACYS D  70       45.125 -14.274   0.358  0.80  8.28      DAC2 C
ANISOU18817  CB ACYS D  70      1270    944    931     54    226     13  DAC2 C
ATOM  18818  SG ACYS D  70       45.858 -12.700  -0.171  0.80  8.08      DAC2 S
ANISOU18818  SG ACYS D  70      1209    904    955    122    322     36  DAC2 S
ATOM  18819  CA ACYS D  95       47.419  -6.957  -4.074  0.80  8.03      DAC2 C
ANISOU18819  CA ACYS D  95      1287    947    816    235     86    -49  DAC2 C
ATOM  18820  CB ACYS D  95       48.291  -8.211  -4.082  0.80  8.40      DAC2 C
ANISOU18820  CB ACYS D  95      1261    986    945    306     96    -39  DAC2 C
ATOM  18821  SG ACYS D  95       47.954  -9.384  -5.446  0.80  7.55      DAC2 S
ANISOU18821  SG ACYS D  95      1054    936    878     76    125    -10  DAC2 S
ATOM  18842  CA ACYS D 153       49.410 -15.598  -6.737  0.80  8.60      DAC2 C
ANISOU18842  CA ACYS D 153       958   1044   1267    129    -14   -120  DAC2 C
ATOM  18843  CB ACYS D 153       47.913 -15.986  -6.573  0.80  8.07      DAC2 C
```

```
ANISOU18843  CB ACYS D 153       981    993   1093    212    -16     62  DAC2 C
ATOM  18844  SG ACYS D 153       46.689 -14.674  -6.793  0.80  7.65       DAC2 S
ANISOU18844  SG ACYS D 153      1095    870    941    148     70     39  DAC2 S
ATOM  18858  N  ASER D 188       48.420 -17.568  -2.209  0.80  8.22       DAC2 N
ANISOU18858  N  ASER D 188       969   1001   1155     81     55     -1  DAC2 N
ATOM  18859  CA ASER D 188       47.088 -17.239  -1.690  0.80  8.72       DAC2 C
ANISOU18859  CA ASER D 188      1189   1007   1119    252     88    -28  DAC2 C
ATOM  18860  C  ASER D 188       46.058 -18.368  -1.770  0.80  8.74       DAC2 C
ANISOU18860  C  ASER D 188      1090   1039   1193    313    171     -2  DAC2 C
ATOM  18861  CB ASER D 188       46.594 -15.918  -2.305  0.80  8.95       DAC2 C
ANISOU18861  CB ASER D 188      1481    965    955    225     59     43  DAC2 C
ATOM  18862  OG ASER D 188       46.670 -15.916  -3.691  0.80  7.90       DAC2 O
ANISOU18862  OG ASER D 188      1276    866    859    304    132     46  DAC2 O
ATOM  18944  S1 A1CL D7498       45.573 -11.306  -6.887  0.80  7.87       DAC2 S2-
ANISOU18944  S1 A1CL D7498      1113    865   1011     79    148   -102  DAC2 S2-
ATOM  18945  S2AA1CL D7498       46.536  -7.915  -8.726  0.80  7.83       DAC2 S2-
ANISOU18945  S2AA1CL D7498       964   1048    965     94    104     -6  DAC2 S2-
ATOM  18946  S2BA1CL D7498       44.117 -13.811  -3.541  0.80  7.92       DAC2 S2-
ANISOU18946  S2BA1CL D7498      1195    815   1002     84    169    -33  DAC2 S2-
ATOM  18947  S3AA1CL D7498       43.355  -9.510  -9.281  0.80  7.26       DAC2 S2-
ANISOU18947  S3AA1CL D7498       864    942    952    113     12    -57  DAC2 S2-
ATOM  18948  S3BA1CL D7498       47.663 -12.684  -3.693  0.80  8.11       DAC2 S2-
ANISOU18948  S3BA1CL D7498      1212    917    954     53    207    -16  DAC2 S2-
ATOM  18949  S4AA1CL D7498       44.115  -7.817  -6.117  0.80  7.67       DAC2 S2-
ANISOU18949  S4AA1CL D7498      1092    884    940     51    100    -97  DAC2 S2-
ATOM  18950  S4BA1CL D7498       44.990 -10.327  -3.151  0.80  7.96       DAC2 S2-
ANISOU18950  S4BA1CL D7498      1285    776    961     94    294    -20  DAC2 S2-
ATOM  18951 FE1 A1CL D7498       44.294  -7.582  -8.367  0.80  7.21       DAC2Fe2+
ANISOU18951 FE1 A1CL D7498       914    894    932     85     15      5  DAC2Fe2+
ATOM  18952 FE2 A1CL D7498       46.483 -11.060  -4.770  0.80  7.82       DAC2Fe2+
ANISOU18952 FE2 A1CL D7498      1119    896    957    127    184    -22  DAC2Fe2+
ATOM  18953 FE3 A1CL D7498       45.590 -10.025  -8.875  0.80  7.43       DAC2Fe2+
ANISOU18953 FE3 A1CL D7498       924    933    967     93     34    -48  DAC2Fe2+
ATOM  18954 FE4 A1CL D7498       43.706  -9.885  -7.020  0.80  7.44       DAC2Fe2+
ANISOU18954 FE4 A1CL D7498      1035    847    946     71    170    -59  DAC2Fe2+
ATOM  18955 FE5 A1CL D7498       45.659 -12.421  -2.535  0.80  8.90       DAC2Fe2+
ANISOU18955 FE5 A1CL D7498      1560    895    927    170    396    -28  DAC2Fe2+
ATOM  18956 FE6 A1CL D7498       46.204 -14.292  -4.540  0.80  7.31       DAC2Fe2+
ANISOU18956 FE6 A1CL D7498      1007    809    961    122    153    -41  DAC2Fe2+
ATOM  18957 FE7 A1CL D7498       43.282 -11.708  -3.857  0.80  6.80       DAC2Fe2+
ANISOU18957 FE7 A1CL D7498       883    786    914     80    104    -37  DAC2Fe2+
ATOM  18958 FE8 A1CL D7498       46.069  -8.875  -6.672  0.80  7.76       DAC2Fe2+
ANISOU18958 FE8 A1CL D7498      1148    882    919     41    125    -31  DAC2Fe2+
```

```
QM system with H atoms
PN state
ATOM      1  H   ???     1       44.804  -3.777 -10.487
ATOM      2  C   ???     1       45.060  -4.825 -10.171
ATOM      3  H   ???     1       45.245  -5.438 -11.076
ATOM      4  H   ???     1       45.995  -4.816  -9.575
ATOM      5  S   ???     1       43.650  -5.509  -9.163
ATOM      6  H   ???     1       41.837 -13.328  -1.234
ATOM      7  C   ???     1       42.153 -12.282  -1.053
ATOM      8  H   ???     1       42.213 -12.128   0.040
ATOM      9  H   ???     1       43.180 -12.192  -1.461
ATOM     10  C   ???     1       41.258 -11.206  -1.625
ATOM     11  O   ???     1       40.611 -10.414  -0.918
ATOM     12  N   ???     1       41.264 -11.146  -2.986
ATOM     13  H   ???     1       41.973 -11.705  -3.491
ATOM     14  C   ???     1       40.866  -9.923  -3.659
ATOM     15  H   ???     1       41.527  -9.092  -3.340
ATOM     16  C   ???     1       40.894 -10.051  -5.199
ATOM     17  H   ???     1       39.904 -10.389  -5.565
ATOM     18  H   ???     1       41.114  -9.061  -5.637
ATOM     19  S   ???     1       42.084 -11.275  -5.905
ATOM     20  H   ???     1       39.821  -9.685  -3.370
ATOM     21  H   ???     1       45.299 -13.149 -11.264
ATOM     22  C   ???     1       46.224 -12.868 -10.700
ATOM     23  H   ???     1       46.153 -13.287  -9.677
ATOM     24  H   ???     1       47.071 -13.389 -11.204
ATOM     25  S   ???     1       46.585 -11.077 -10.643
ATOM     26  H   ???     1       45.149 -14.464   1.465
ATOM     27  C   ???     1       45.151 -14.241   0.347
ATOM     28  H   ???     1       45.748 -15.061  -0.099
ATOM     29  H   ???     1       44.106 -14.395   0.003
ATOM     30  S   ???     1       45.763 -12.609  -0.229
ATOM     31  H   ???     1       47.649  -7.308  -4.084
ATOM     32  C   ???     1       48.261  -8.224  -4.099
ATOM     33  H   ???     1       49.333  -7.932  -4.079
ATOM     34  H   ???     1       48.031  -8.801  -3.183
ATOM     35  S   ???     1       47.973  -9.277  -5.580
ATOM     36  H   ???     1       48.916 -15.631  -6.735
ATOM     37  C   ???     1       47.799 -15.782  -6.686
ATOM     38  H   ???     1       47.581 -16.638  -7.366
ATOM     39  H   ???     1       47.565 -16.113  -5.656
ATOM     40  S   ???     1       46.756 -14.366  -7.162
ATOM     41  H   ???     1       48.098 -17.485  -2.104
ATOM     42  C   ???     1       47.073 -17.221  -1.734
ATOM     43  H   ???     1       47.194 -16.936  -0.666
ATOM     44  C   ???     1       46.511 -16.018  -2.497
ATOM     45  H   ???     1       45.592 -15.654  -1.994
ATOM     46  H   ???     1       47.230 -15.173  -2.483
ATOM     47  O   ???     1       46.199 -16.415  -3.824
ATOM     48  H   ???     1       45.714 -15.640  -4.224
ATOM     49  H   ???     1       46.333 -18.050  -1.768
ATOM     50  S   ???     1       45.518 -11.344  -6.836
ATOM     51 FE   ???     1       44.125  -7.620  -8.466
ATOM     52 FE   ???     1       43.610  -9.911  -7.107
ATOM     53 FE   ???     1       45.404 -10.052  -8.939
ATOM     54 FE   ???     1       45.999 -13.160  -5.291
ATOM     55 FE   ???     1       44.200 -11.557  -4.809
ATOM     56 FE   ???     1       45.891  -8.937  -6.744
ATOM     57 FE   ???     1       45.673 -12.418  -2.548
ATOM     58 FE   ???     1       46.595 -10.912  -4.624
ATOM     59  S   ???     1       46.423  -7.961  -8.775
ATOM     60  S   ???     1       44.172 -13.722  -3.856
ATOM     61  S   ???     1       43.143  -9.560  -9.344
ATOM     62  S   ???     1       47.666 -12.741  -3.681
ATOM     63  S   ???     1       43.937  -7.844  -6.145
ATOM     64  S   ???     1       44.987 -10.263  -3.072
END
```

```
QM system with H atoms
P2+ state
ATOM      1  H   ???     1       44.800  -3.783 -10.480
ATOM      2  C   ???     1       45.051  -4.832 -10.156
ATOM      3  H   ???     1       45.229  -5.450 -11.059
ATOM      4  H   ???     1       45.988  -4.821  -9.567
ATOM      5  S   ???     1       43.654  -5.517  -9.140
ATOM      6  H   ???     1       41.817 -13.288  -1.207
ATOM      7  C   ???     1       42.095 -12.220  -0.987
ATOM      8  H   ???     1       41.996 -12.080   0.110
ATOM      9  H   ???     1       43.175 -12.142  -1.231
ATOM     10  C   ???     1       41.315 -11.096  -1.668
ATOM     11  O   ???     1       40.620 -10.299  -0.985
ATOM     12  N   ???     1       41.519 -11.032  -2.999
ATOM     13  C   ???     1       40.884  -9.925  -3.692
ATOM     14  H   ???     1       41.471  -8.992  -3.525
ATOM     15  C   ???     1       40.797 -10.177  -5.207
ATOM     16  H   ???     1       39.884 -10.764  -5.445
ATOM     17  H   ???     1       40.754  -9.225  -5.767
ATOM     18  S   ???     1       42.218 -11.172  -5.832
ATOM     19  H   ???     1       39.826  -9.686  -3.382
ATOM     20  H   ???     1       45.309 -13.154 -11.237
ATOM     21  C   ???     1       46.237 -12.891 -10.640
ATOM     22  H   ???     1       46.098 -13.286  -9.615
ATOM     23  H   ???     1       47.073 -13.464 -11.100
ATOM     24  S   ???     1       46.731 -11.126 -10.561
ATOM     25  H   ???     1       45.139 -14.479   1.473
ATOM     26  C   ???     1       45.127 -14.273   0.359
ATOM     27  H   ???     1       45.651 -15.130  -0.102
ATOM     28  H   ???     1       44.069 -14.334   0.032
ATOM     29  S   ???     1       45.860 -12.698  -0.170
ATOM     30  H   ???     1       47.661  -7.306  -4.077
ATOM     31  C   ???     1       48.289  -8.212  -4.082
ATOM     32  H   ???     1       49.354  -7.897  -4.132
ATOM     33  H   ???     1       48.120  -8.745  -3.127
ATOM     34  S   ???     1       47.956  -9.385  -5.446
ATOM     35  H   ???     1       48.992 -15.704  -6.695
ATOM     36  C   ???     1       47.910 -15.983  -6.579
ATOM     37  H   ???     1       47.731 -16.793  -7.325
ATOM     38  H   ???     1       47.806 -16.428  -5.569
ATOM     39  S   ???     1       46.685 -14.671  -6.797
ATOM     40  H   ???     1       48.100 -17.488  -2.084
ATOM     41  C   ???     1       47.088 -17.238  -1.692
ATOM     42  H   ???     1       47.227 -17.071  -0.596
ATOM     43  C   ???     1       46.593 -15.919  -2.309
ATOM     44  H   ???     1       45.544 -15.749  -1.970
ATOM     45  H   ???     1       47.193 -15.087  -1.862
ATOM     46  O   ???     1       46.668 -15.918  -3.695
ATOM     47  H   ???     1       46.341 -18.059  -1.749
ATOM     48  S   ???     1       45.574 -11.308  -6.887
ATOM     49 FE   ???     1       44.294  -7.582  -8.369
ATOM     50 FE   ???     1       46.484 -11.062  -4.771
ATOM     51 FE   ???     1       45.590 -10.026  -8.875
ATOM     52 FE   ???     1       43.707  -9.885  -7.021
ATOM     53 FE   ???     1       45.659 -12.422  -2.537
ATOM     54 FE   ???     1       46.204 -14.291  -4.541
ATOM     55 FE   ???     1       43.283 -11.708  -3.857
ATOM     56 FE   ???     1       46.068  -8.876  -6.673
ATOM     57  S   ???     1       46.538  -7.915  -8.728
ATOM     58  S   ???     1       44.115 -13.813  -3.542
ATOM     59  S   ???     1       43.354  -9.509  -9.283
ATOM     60  S   ???     1       47.664 -12.685  -3.694
ATOM     61  S   ???     1       44.114  -7.816  -6.117
ATOM     62  S   ???     1       44.992 -10.326  -3.151
END
```

b)

```
REMARK Quantum-refined structure of 6CDK, obtained with wA=1 and 50/50 occupancy
ATOM   8272  CA  CYS    62       65.040 -20.740   0.062  0.50 24.65      CAC1 C
ATOM   8273  CB  CYS    62       64.769 -22.200   0.497  0.50 19.77      CAC1 C
ATOM   8274  SG  CYS    62       63.402 -22.885  -0.550  0.50 25.02      CAC1 S
ATOM   8454  N   GLY    87       55.296 -30.855   0.787  0.50 21.85      CAC1 N
ATOM   8455  CA  GLY    87       55.212 -29.469   1.235  0.50 23.07      CAC1 C
ATOM   8456  C   GLY    87       55.436 -28.410   0.165  0.50 21.72      CAC1 C
ATOM   8457  O   GLY    87       54.561 -27.597  -0.175  0.50 17.63      CAC1 O
ATOM   8458  N   CYS    88       56.694 -28.393  -0.373  0.50 22.37      CAC1 N
ATOM   8459  CA  CYS    88       57.214 -27.191  -1.015  0.50 23.17      CAC1 C
ATOM   8460  C   CYS    88       56.330 -26.820  -2.219  0.50 23.56      CAC1 C
ATOM   8461  CB  CYS    88       58.551 -27.577  -1.693  0.50 25.55      CAC1 C
ATOM   8462  SG  CYS    88       59.724 -28.562  -0.654  0.50 30.34      CAC1 S
ATOM   8977  CA  CYS   154       66.028 -30.532   0.102  0.50 27.96      CAC1 C
ATOM   8978  CB  CYS   154       65.627 -30.190   1.567  0.50 26.72      CAC1 C
ATOM   8979  SG  CYS   154       65.661 -28.385   1.945  0.50 25.86      CAC1 S
ATOM  12176  CA  CYS    70       53.520 -31.782   4.970  0.50 25.47      DAC1 C
ATOM  12177  CB  CYS    70       54.962 -31.505   4.458  0.50 40.04      DAC1 C
ATOM  12178  SG  CYS    70       55.596 -29.818   4.780  0.50 22.98      DAC1 S
ATOM  12356  CA  CYS    95       59.922 -24.186   4.965  0.50 24.05      DAC1 C
ATOM  12357  CB  CYS    95       60.246 -25.436   5.796  0.50 24.29      DAC1 C
ATOM  12358  SG  CYS    95       61.506 -26.532   5.009  0.50 24.83      DAC1 S
ATOM  12809  CA  CYS   153       63.042 -32.869   5.813  0.50 43.17      DAC1 C
ATOM  12810  CB  CYS   153       62.424 -33.128   4.402  0.50 64.16      DAC1 C
ATOM  12811  SG  CYS   153       62.259 -31.693   3.286  0.50 34.46      DAC1 S
ATOM  13079  N   SER   188       58.443 -34.774   6.496  0.50 27.64      DAC1 N
ATOM  13080  CA  SER   188       57.508 -34.416   5.410  0.50 28.04      DAC1 C
ATOM  13081  C   SER   188       57.211 -35.622   4.493  0.50 29.31      DAC1 C
ATOM  13082  CB  SER   188       57.960 -33.117   4.666  0.50 34.17      DAC1 C
ATOM  13083  OG  SER   188       59.278 -33.055   4.261  0.50 33.50      DAC1 O
ATOM  15856 FE   FE    520       57.771 -29.697   4.004  0.50 22.05      DAC1Fe2+
ATOM  15857 FE   FE    521       62.802 -24.893   0.321  0.50 19.76      DAC1Fe2+
ATOM  15858 FE   FE    522       63.667 -27.316   1.383  0.50 18.81      DAC1Fe2+
ATOM  15859 FE   FE    523       59.353 -28.799   1.793  0.50 33.75      DAC1Fe2+
ATOM  15860  S   S     524       61.797 -28.635   2.184  0.50 27.90      DAC1 S2-
ATOM  15861  S   S     525       63.936 -25.208   2.358  0.50 21.70      DAC1 S2-
ATOM  15862  S   S     526       63.242 -26.858  -0.880  0.50 64.04      DAC1 S2-
ATOM  15863  S   S     527       60.566 -25.070   1.007  0.50 26.58      DAC1 S2-
ATOM  15864 FE   FE    528       61.840 -26.204   2.597  0.50 34.75      DAC1Fe2+
ATOM  15865 FE   FE    529       59.975 -31.328   3.628  0.50 33.19      DAC1Fe2+
ATOM  15866 FE   FE    530       61.313 -27.164   0.369  0.50 23.61      DAC1Fe2+
ATOM  15867 FE   FE    531       60.180 -28.269   4.081  0.50 32.52      DAC1Fe2+
ATOM  15868  S   S     532       58.315 -30.852   2.010  0.50 74.80      DAC1 S2-
ATOM  15869  S   S     533       59.519 -29.979   5.506  0.50 68.26      DAC1 S2-
ATOM  15870  S   S     534       58.119 -27.504   3.233  0.50 24.15      DAC1 S2-
ATOM  16612  CA  CYS    62       65.040 -20.739   0.063  0.50 24.79      CAC2 C
ATOM  16613  CB  CYS    62       64.771 -22.197   0.501  0.50 19.76      CAC2 C
ATOM  16614  SG  CYS    62       63.364 -22.848  -0.505  0.50 25.05      CAC2 S
ATOM  16615  N   GLY    87       55.286 -30.844   0.788  0.50 21.84      CAC2 N
ATOM  16616  CA  GLY    87       55.179 -29.445   1.219  0.50 23.13      CAC2 C
ATOM  16617  C   GLY    87       55.477 -28.384   0.142  0.50 21.71      CAC2 C
ATOM  16618  O   GLY    87       54.541 -27.739  -0.391  0.50 17.71      CAC2 O
ATOM  16619  N   CYS    88       56.797 -28.239  -0.111  0.50 22.19      CAC2 N
ATOM  16620  CA  CYS    88       57.247 -27.175  -1.001  0.50 23.21      CAC2 C
ATOM  16621  C   CYS    88       56.335 -26.819  -2.219  0.50 23.44      CAC2 C
ATOM  16622  CB  CYS    88       58.496 -27.735  -1.734  0.50 25.74      CAC2 C
ATOM  16623  SG  CYS    88       59.692 -28.554  -0.575  0.50 30.80      CAC2 S
ATOM  16624  CA  CYS   154       66.031 -30.530   0.099  0.50 27.94      CAC2 C
ATOM  16625  CB  CYS   154       65.643 -30.169   1.562  0.50 26.70      CAC2 C
ATOM  16626  SG  CYS   154       65.717 -28.353   1.891  0.50 25.71      CAC2 S
ATOM  16627  CA  CYS    70       53.525 -31.778   4.967  0.50 25.32      DAC2 C
ATOM  16628  CB  CYS    70       54.974 -31.483   4.451  0.50 38.36      DAC2 C
ATOM  16629  SG  CYS    70       55.683 -29.867   4.927  0.50 22.99      DAC2 S
ATOM  16630  CA  CYS    95       59.918 -24.183   4.959  0.50 24.05      DAC2 C
ATOM  16631  CB  CYS    95       60.225 -25.454   5.775  0.50 24.29      DAC2 C
ATOM  16632  SG  CYS    95       61.327 -26.690   4.951  0.50 24.55      DAC2 S
ATOM  16633  CA  CYS   153       63.043 -32.870   5.814  0.50 42.38      DAC2 C
ATOM  16634  CB  CYS   153       62.423 -33.142   4.400  0.50 65.32      DAC2 C
ATOM  16635  SG  CYS   153       62.251 -31.748   3.235  0.50 32.68      DAC2 S
```

ATOM	16636	N	SER	188	58.441	-34.774	6.497	0.50	27.69	DAC2 N
ATOM	16637	CA	SER	188	57.504	-34.421	5.415	0.50	28.09	DAC2 C
ATOM	16638	C	SER	188	57.210	-35.623	4.496	0.50	29.30	DAC2 C
ATOM	16639	CB	SER	188	57.958	-33.122	4.686	0.50	34.08	DAC2 C
ATOM	16640	OG	SER	188	59.290	-33.079	4.303	0.50	33.48	DAC2 O
ATOM	16641	FE	FE	520	60.251	-28.324	3.748	0.50	29.14	DAC2Fe2+
ATOM	16642	FE	FE	521	63.681	-27.308	1.426	0.50	18.84	DAC2Fe2+
ATOM	16643	FE	FE	522	62.816	-24.872	0.443	0.50	19.71	DAC2Fe2+
ATOM	16644	FE	FE	523	61.277	-27.162	0.419	0.50	23.61	DAC2Fe2+
ATOM	16645	S	S	524	61.852	-28.644	2.151	0.50	27.83	DAC2 S2-
ATOM	16646	S	S	525	63.903	-25.208	2.433	0.50	21.18	DAC2 S2-
ATOM	16647	S	S	526	63.230	-26.776	-0.813	0.50	69.58	DAC2 S2-
ATOM	16648	S	S	527	60.594	-25.047	1.086	0.50	26.61	DAC2 S2-
ATOM	16649	FE	FE	528	59.946	-31.408	3.612	0.50	33.30	DAC2Fe2+
ATOM	16650	FE	FE	529	58.205	-28.888	1.208	0.50	20.12	DAC2Fe2+
ATOM	16651	FE	FE	530	57.860	-29.612	3.895	0.50	22.45	DAC2Fe2+
ATOM	16652	FE	FE	531	61.813	-26.253	2.661	0.50	39.99	DAC2Fe2+
ATOM	16653	S	S	532	58.227	-30.989	2.039	0.50	69.19	DAC2 S2-
ATOM	16654	S	S	533	59.685	-29.865	5.330	0.50	64.52	DAC2 S2-
ATOM	16655	S	S	534	58.174	-27.511	3.054	0.50	24.73	DAC2 S2-

```
QM system with H atoms
P1+ state
REMARK Energies (QM/MM, QM) =  -15769.486630 -15925.930339 H
REMARK /lunarc/nobackup/projects/snic2019-35-66/Ulf/Cqx2qm/Cdk6/Correct/1
REMARK Tue Sep  1 15:08:00 CEST 2020
ATOM      1  H   ???     1      64.965 -21.147   0.184
ATOM      2  C   ???     1      64.770 -22.201   0.498
ATOM      3  H   ???     1      65.701 -22.791   0.382
ATOM      4  H   ???     1      64.489 -22.228   1.568
ATOM      5  S   ???     1      63.402 -22.886  -0.550
ATOM      6  H   ???     1      55.276 -30.527   0.894
ATOM      7  C   ???     1      55.213 -29.469   1.235
ATOM      8  H   ???     1      54.211 -29.302   1.670
ATOM      9  H   ???     1      55.955 -29.358   2.051
ATOM     10  C   ???     1      55.436 -28.411   0.166
ATOM     11  O   ???     1      54.561 -27.598  -0.176
ATOM     12  N   ???     1      56.694 -28.394  -0.374
ATOM     13  H   ???     1      57.416 -28.975   0.090
ATOM     14  C   ???     1      57.214 -27.191  -1.015
ATOM     15  H   ???     1      57.331 -26.350  -0.306
ATOM     16  C   ???     1      58.551 -27.577  -1.694
ATOM     17  H   ???     1      58.331 -28.218  -2.570
ATOM     18  H   ???     1      59.073 -26.664  -2.036
ATOM     19  S   ???     1      59.724 -28.562  -0.654
ATOM     20  H   ???     1      56.573 -26.922  -1.889
ATOM     21  H   ???     1      65.917 -30.438   0.510
ATOM     22  C   ???     1      65.628 -30.190   1.567
ATOM     23  H   ???     1      64.622 -30.608   1.769
ATOM     24  H   ???     1      66.349 -30.711   2.235
ATOM     25  S   ???     1      65.661 -28.386   1.945
ATOM     26  H   ???     1      53.921 -31.706   4.828
ATOM     27  C   ???     1      54.963 -31.505   4.459
ATOM     28  H   ???     1      55.603 -32.263   4.944
ATOM     29  H   ???     1      54.974 -31.720   3.370
ATOM     30  S   ???     1      55.596 -29.818   4.780
ATOM     31  H   ???     1      60.013 -24.534   5.197
ATOM     32  C   ???     1      60.247 -25.436   5.797
ATOM     33  H   ???     1      60.637 -25.114   6.785
ATOM     34  H   ???     1      59.306 -25.999   5.944
ATOM     35  S   ???     1      61.507 -26.532   5.009
ATOM     36  H   ???     1      62.871 -32.941   5.421
ATOM     37  C   ???     1      62.425 -33.128   4.403
ATOM     38  H   ???     1      63.074 -33.899   3.924
ATOM     39  H   ???     1      61.426 -33.577   4.574
ATOM     40  S   ???     1      62.259 -31.694   3.286
ATOM     41  H   ???     1      58.218 -34.688   6.235
ATOM     42  C   ???     1      57.509 -34.417   5.410
ATOM     43  H   ???     1      56.534 -34.194   5.904
ATOM     44  C   ???     1      57.961 -33.118   4.666
ATOM     45  H   ???     1      57.266 -32.998   3.797
ATOM     46  H   ???     1      57.714 -32.272   5.359
ATOM     47  O   ???     1      59.279 -33.055   4.262
ATOM     48  H   ???     1      57.293 -35.292   4.745
ATOM     49 FE   ???     1      57.771 -29.697   4.004
ATOM     50 FE   ???     1      62.802 -24.893   0.322
ATOM     51 FE   ???     1      63.668 -27.316   1.384
ATOM     52 FE   ???     1      59.353 -28.799   1.794
ATOM     53  S   ???     1      61.798 -28.635   2.184
ATOM     54  S   ???     1      63.937 -25.209   2.359
ATOM     55  S   ???     1      63.242 -26.859  -0.880
ATOM     56  S   ???     1      60.566 -25.071   1.008
ATOM     57 FE   ???     1      61.840 -26.204   2.597
ATOM     58 FE   ???     1      59.976 -31.329   3.628
ATOM     59 FE   ???     1      61.313 -27.165   0.369
ATOM     60 FE   ???     1      60.181 -28.270   4.082
ATOM     61  S   ???     1      58.316 -30.852   2.011
ATOM     62  S   ???     1      59.520 -29.980   5.506
ATOM     63  S   ???     1      58.119 -27.504   3.234
END
```

```
QM system with H atoms
P2+ state
REMARK Energies (QM/MM, QM) =  -15769.486630 -15925.335435 H
REMARK /lunarc/nobackup/projects/snic2019-35-66/Ulf/Cqx2qm/Cdk6/Correct/1
REMARK Tue Sep  1 15:08:00 CEST 2020
ATOM      1  H   ???     1      64.966 -21.145   0.185
ATOM      2  C   ???     1      64.771 -22.197   0.502
ATOM      3  H   ???     1      65.691 -22.798   0.351
ATOM      4  H   ???     1      64.525 -22.223   1.580
ATOM      5  S   ???     1      63.364 -22.848  -0.506
ATOM      6  H   ???     1      55.261 -30.512   0.891
ATOM      7  C   ???     1      55.180 -29.446   1.220
ATOM      8  H   ???     1      54.137 -29.288   1.564
ATOM      9  H   ???     1      55.840 -29.333   2.100
ATOM     10  C   ???     1      55.477 -28.384   0.143
ATOM     11  O   ???     1      54.541 -27.740  -0.391
ATOM     12  N   ???     1      56.797 -28.240  -0.112
ATOM     13  C   ???     1      57.247 -27.176  -1.002
ATOM     14  H   ???     1      57.504 -26.250  -0.445
ATOM     15  C   ???     1      58.497 -27.736  -1.734
ATOM     16  H   ???     1      58.169 -28.524  -2.440
ATOM     17  H   ???     1      59.033 -26.945  -2.296
ATOM     18  S   ???     1      59.693 -28.554  -0.575
ATOM     19  H   ???     1      56.585 -26.917  -1.886
ATOM     20  H   ???     1      65.924 -30.430   0.506
ATOM     21  C   ???     1      65.644 -30.170   1.562
ATOM     22  H   ???     1      64.632 -30.562   1.778
ATOM     23  H   ???     1      66.362 -30.687   2.235
ATOM     24  S   ???     1      65.717 -28.353   1.891
ATOM     25  H   ???     1      53.928 -31.696   4.824
ATOM     26  C   ???     1      54.974 -31.483   4.451
ATOM     27  H   ???     1      55.597 -32.311   4.836
ATOM     28  H   ???     1      54.953 -31.581   3.347
ATOM     29  S   ???     1      55.683 -29.867   4.927
ATOM     30  H   ???     1      60.003 -24.537   5.186
ATOM     31  C   ???     1      60.225 -25.454   5.775
ATOM     32  H   ???     1      60.706 -25.150   6.729
ATOM     33  H   ???     1      59.265 -25.957   5.997
ATOM     34  S   ???     1      61.328 -26.690   4.951
ATOM     35  H   ???     1      62.871 -32.946   5.422
ATOM     36  C   ???     1      62.423 -33.143   4.401
ATOM     37  H   ???     1      63.071 -33.928   3.943
ATOM     38  H   ???     1      61.428 -33.595   4.586
ATOM     39  S   ???     1      62.251 -31.748   3.235
ATOM     40  H   ???     1      58.216 -34.690   6.236
ATOM     41  C   ???     1      57.504 -34.421   5.416
ATOM     42  H   ???     1      56.530 -34.202   5.912
ATOM     43  C   ???     1      57.958 -33.123   4.687
ATOM     44  H   ???     1      57.286 -32.989   3.804
ATOM     45  H   ???     1      57.714 -32.277   5.377
ATOM     46  O   ???     1      59.290 -33.080   4.304
ATOM     47  H   ???     1      57.291 -35.294   4.748
ATOM     48 FE   ???     1      60.251 -28.325   3.748
ATOM     49 FE   ???     1      63.682 -27.308   1.426
ATOM     50 FE   ???     1      62.816 -24.873   0.443
ATOM     51 FE   ???     1      61.278 -27.163   0.419
ATOM     52  S   ???     1      61.852 -28.645   2.151
ATOM     53  S   ???     1      63.904 -25.209   2.433
ATOM     54  S   ???     1      63.230 -26.776  -0.813
ATOM     55  S   ???     1      60.594 -25.047   1.087
ATOM     56 FE   ???     1      59.947 -31.408   3.613
ATOM     57 FE   ???     1      58.206 -28.888   1.209
ATOM     58 FE   ???     1      57.861 -29.612   3.895
ATOM     59 FE   ???     1      61.814 -26.253   2.661
ATOM     60  S   ???     1      58.227 -30.989   2.039
ATOM     61  S   ???     1      59.686 -29.865   5.330
ATOM     62  S   ???     1      58.174 -27.512   3.054
END
```
